# Supplementary material for: An integrated multi-omics approach reveals polymethoxylated flavonoid biosynthesis in Citrus reticulata cv. Chachiensis
Source: Nat Commun. 2024 May 11;15:3991. doi: 10.1038/s41467-024-48235-y (PMC11088696; doi:10.1038/s41467-024-48235-y)
Supplement: Supplementary file 1 — Supplementary information [file 41467_2024_48235_MOESM1_ESM.pdf]

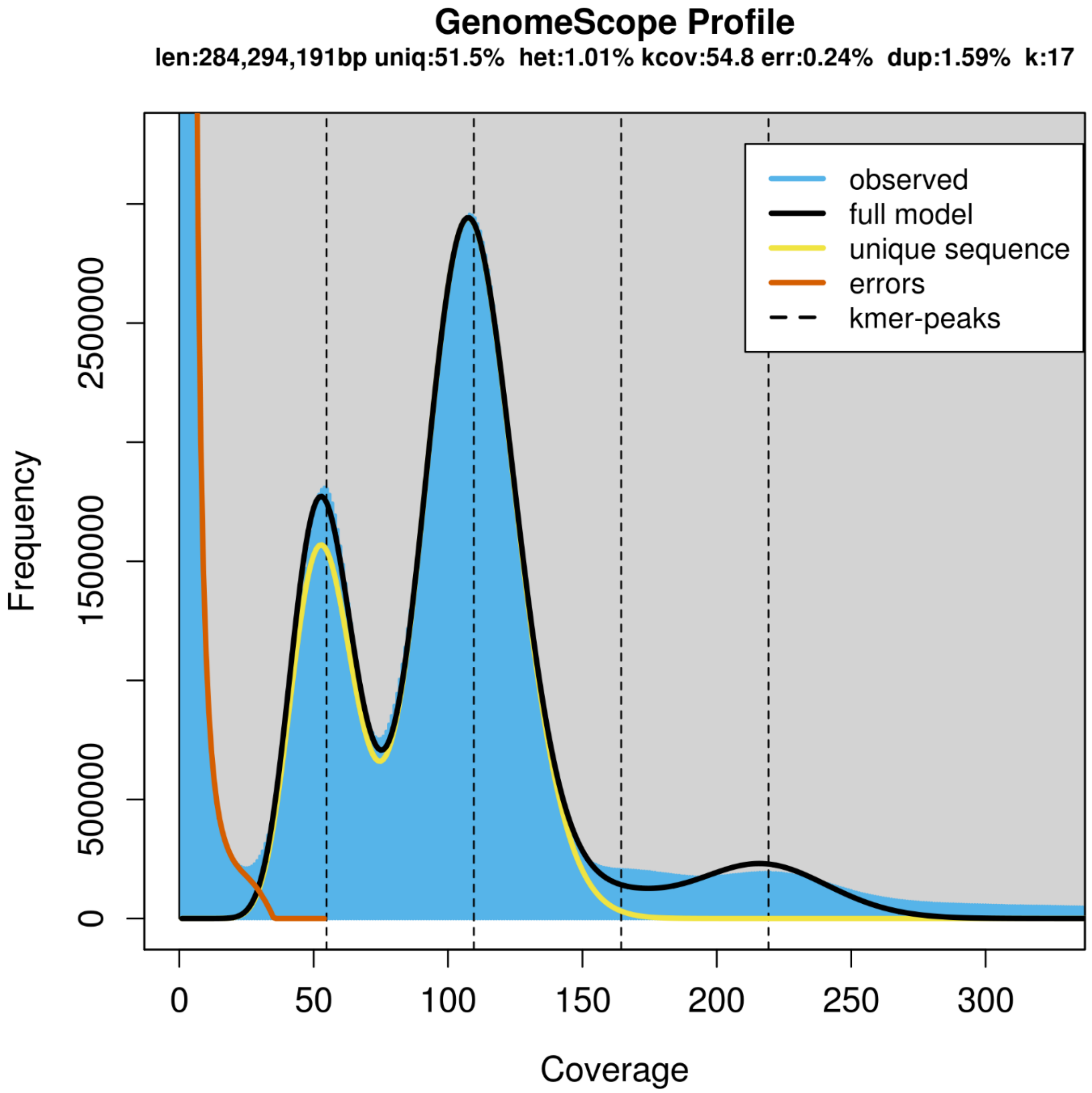

Supplementary Figure 1. The 17-mer depth distribution of CRC.

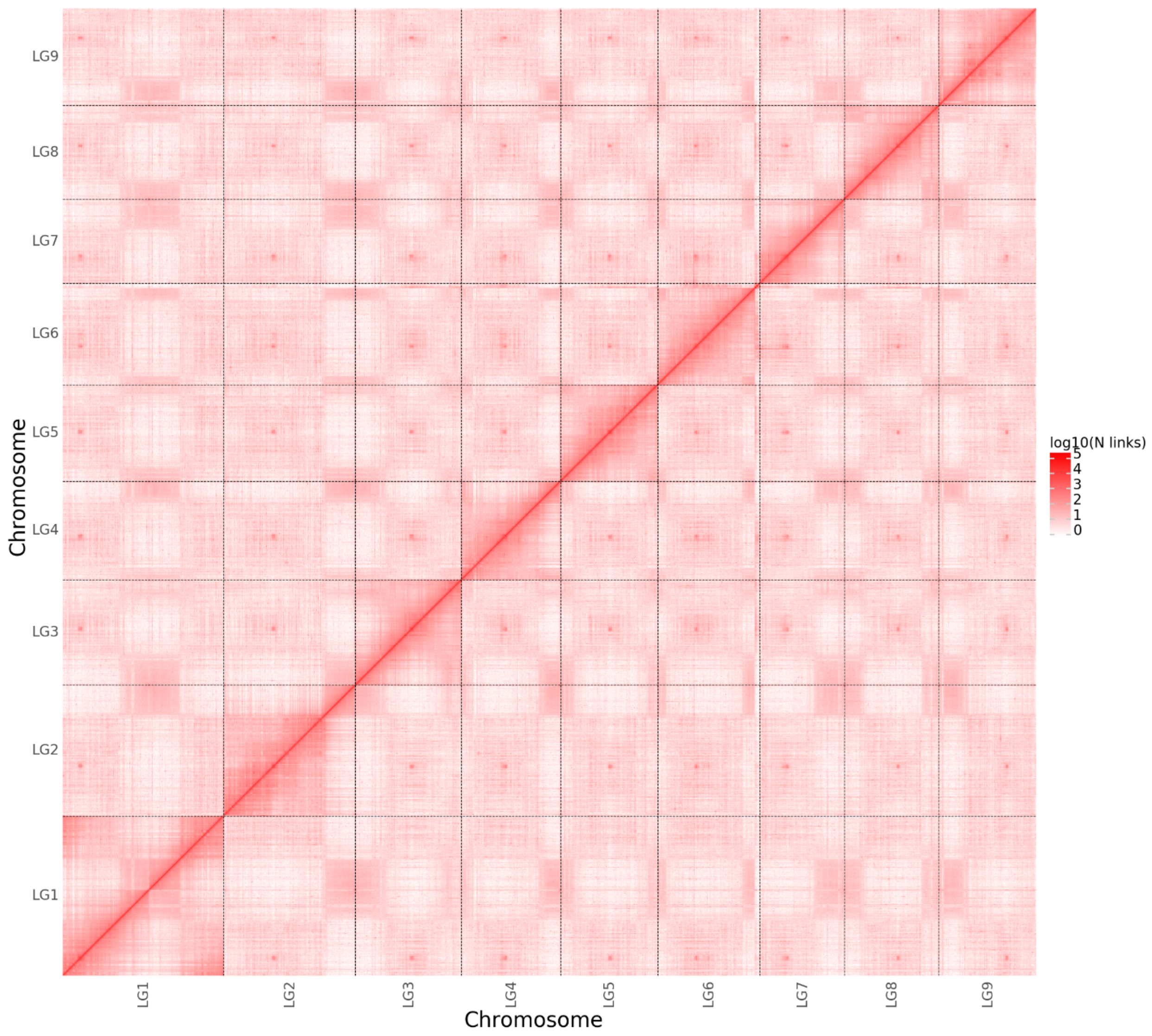

Supplementary Figure 2. Hi-C contact matrix visualization of CRC.

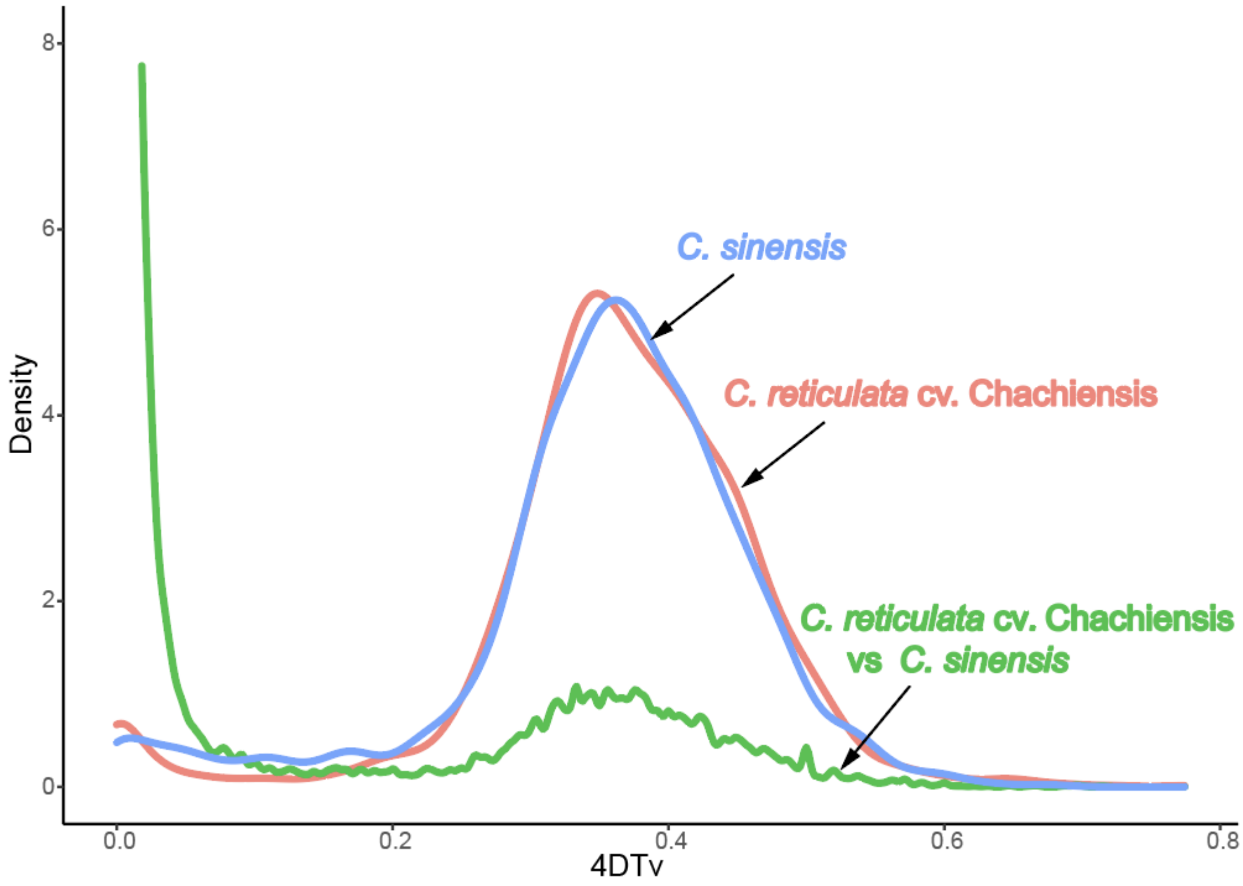

Supplementary Figure 3. Distribution of 4Dtv value of CRC and *C. sinensis.*

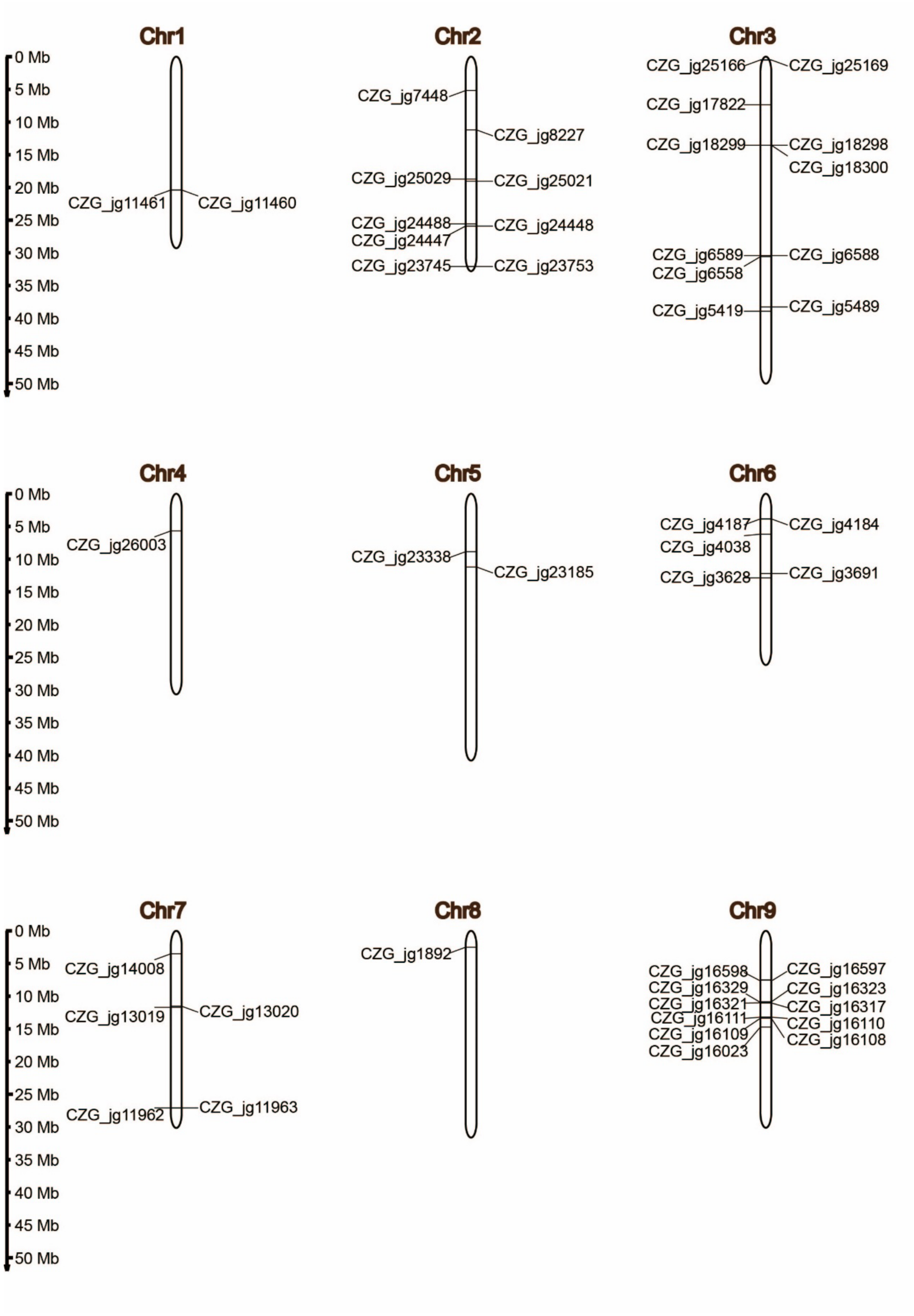

Supplementary Figure 4. Chromosomal distribution of OMT genes in CRC.

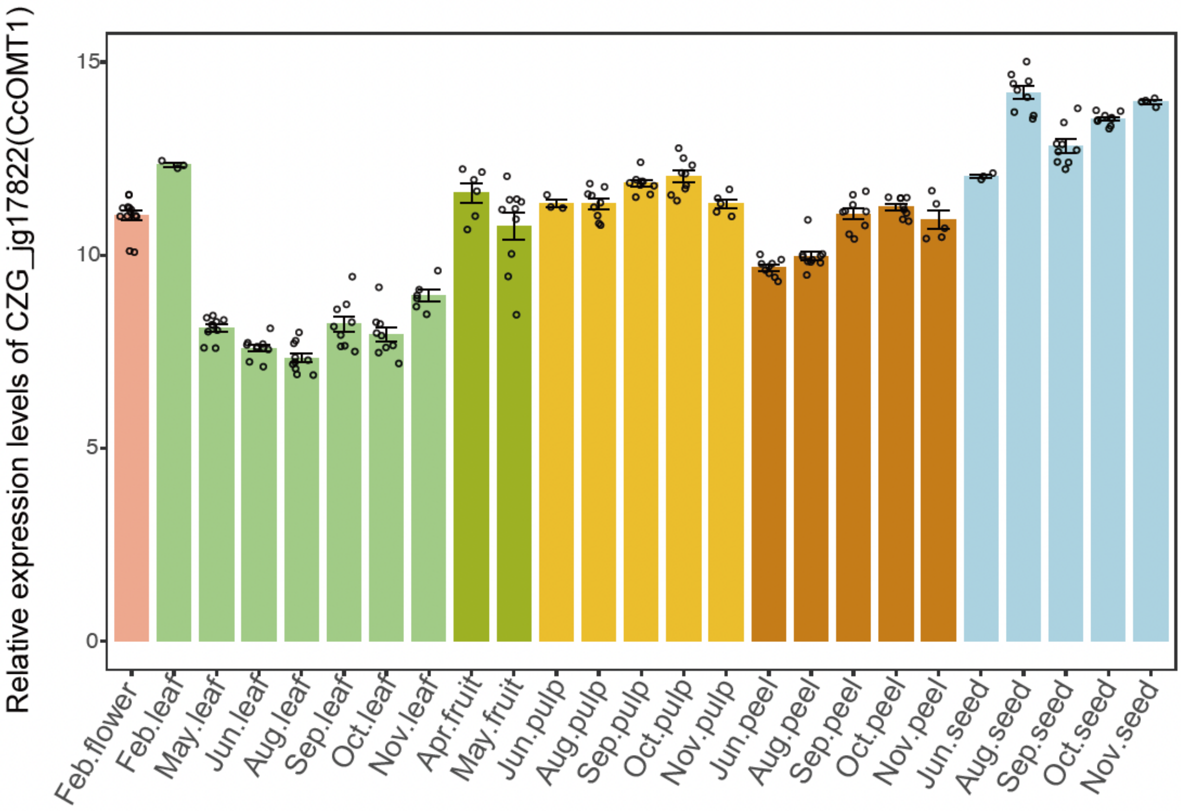

Supplementary Figure 5. Relative expression levels of a COMT gene CZG_jg17822 in different development stages and tissues. Error bars, mean ± s.d. For flowers samples, n=13; for samples of May fruit, May leaf, Jun leaf, Aug leaf and Aug peel, n=10, respectively; for samples of Jun peel, Aug seed, Aug pulp, Sep leaf, Sep peel, Sep pulp, Sep seed, Oct pulp,Oct leaf, Oct peel, and Oct seed, n=9, respectively; for samples of Apr fruit and Nov leaf, n =6, respectively; for samples of Nov peel and Nov pulp, n=4, respectively; for samples of Nov seed, n=4; for samples of Feb leaf, Jun pulp and Jun seed, n=3, respectively. Source data are provided.

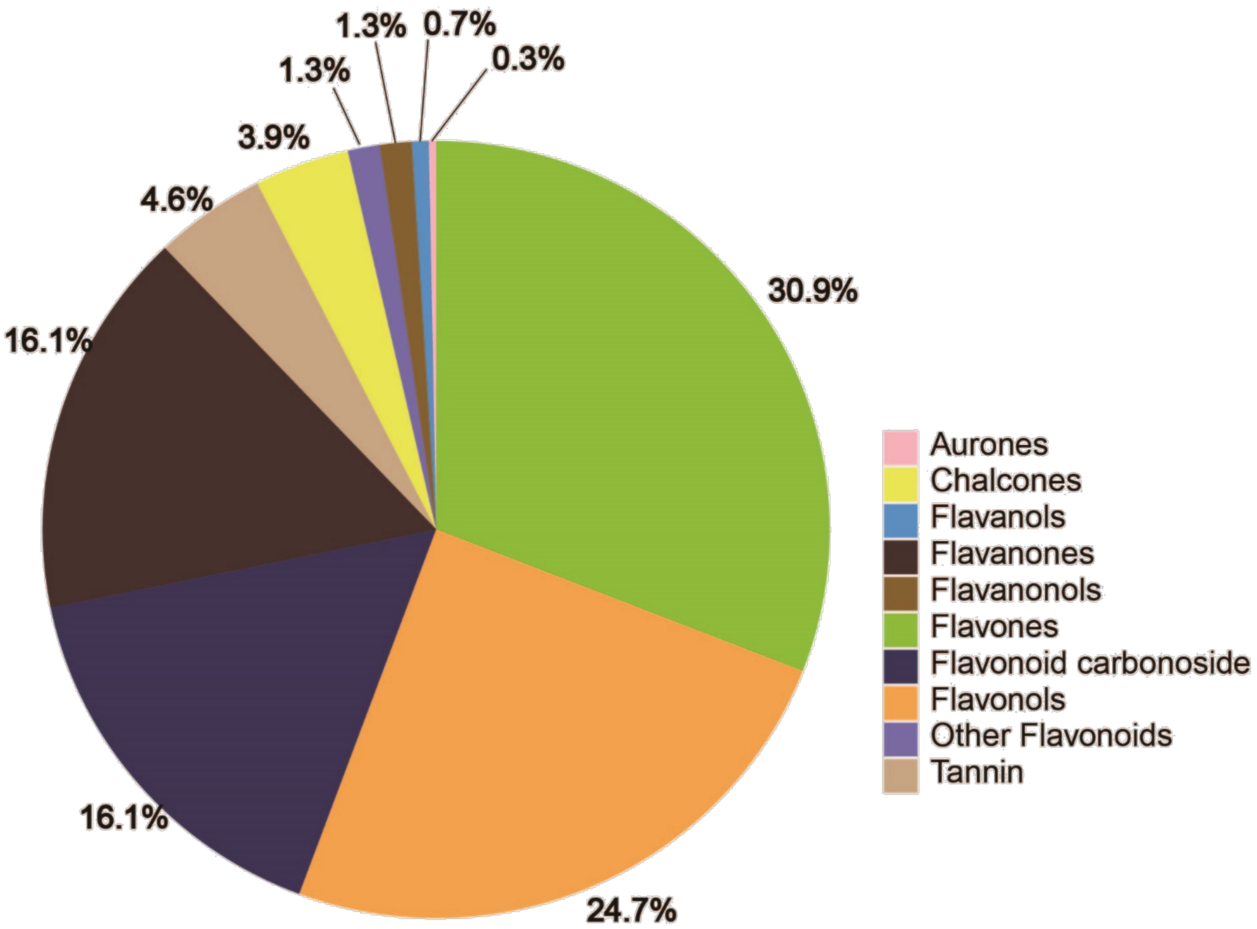

Supplementary Figure 6. The composition of flavonoids in CRC.

a

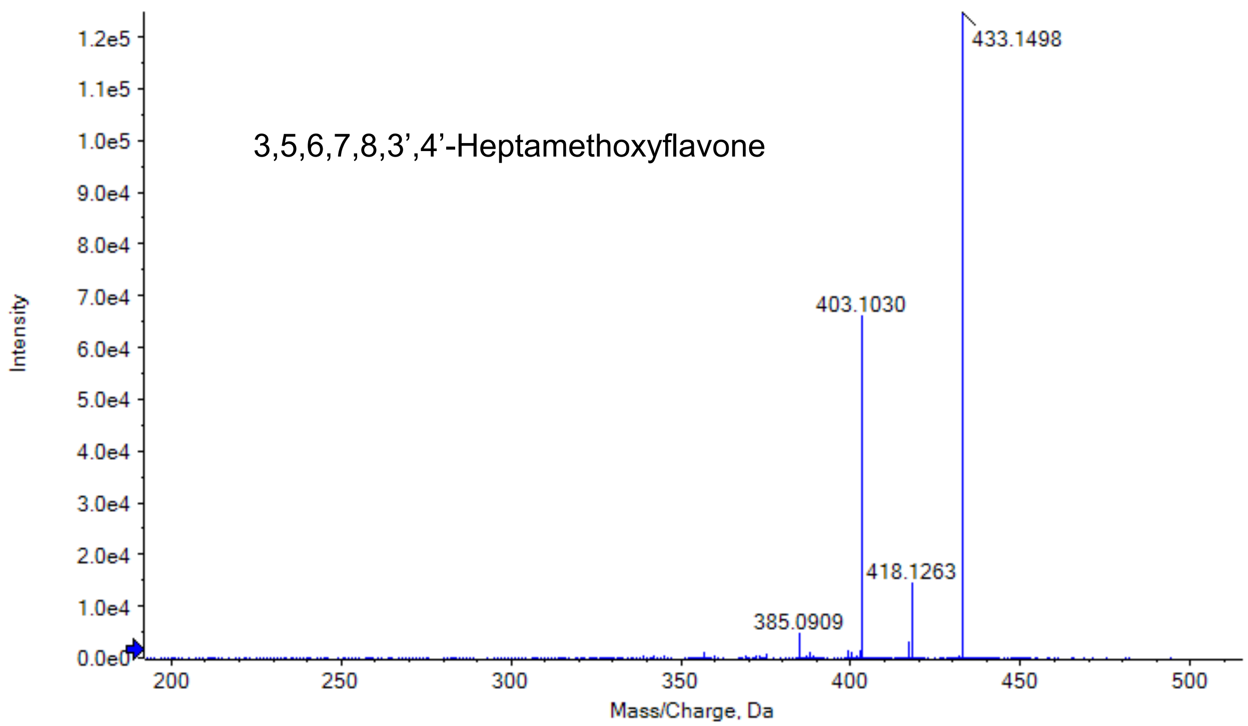

b

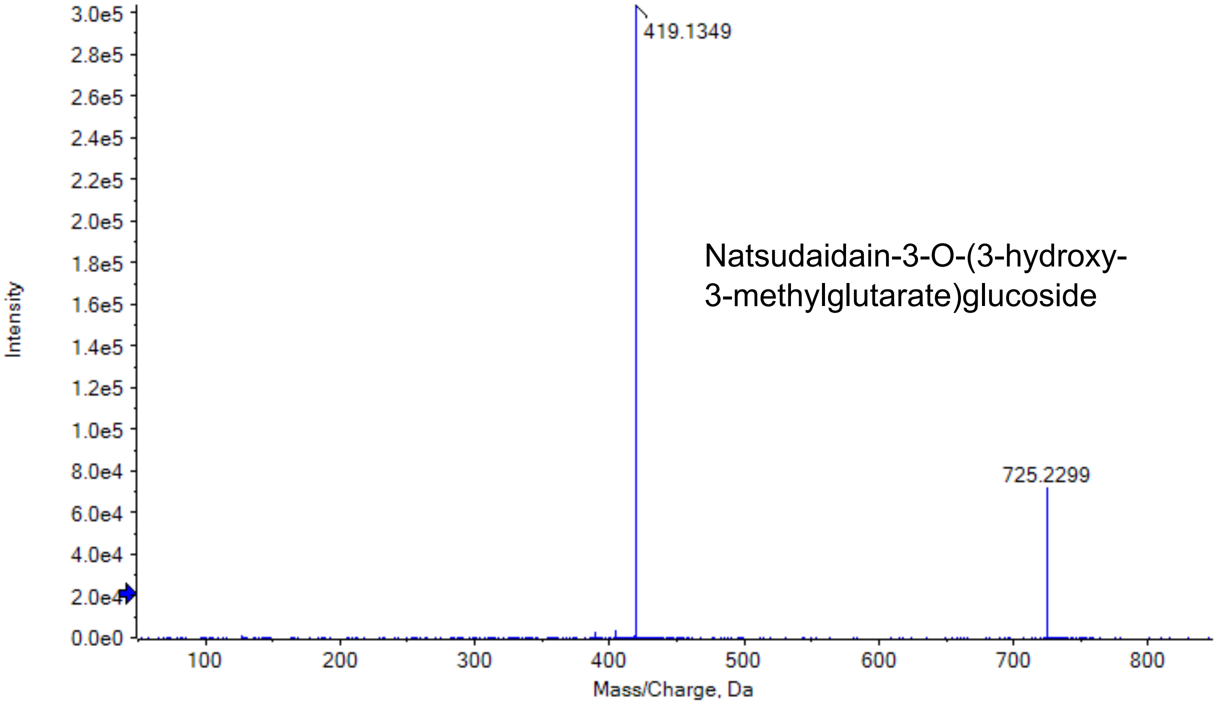

Supplementary Figure 7. Tandem mass spectra for a) 3,5,6,7,8,3',4'-Heptamethoxyflavone and b) Natsudaidain-3-O-(3-hydroxy-3-methylglutarate)glucoside.

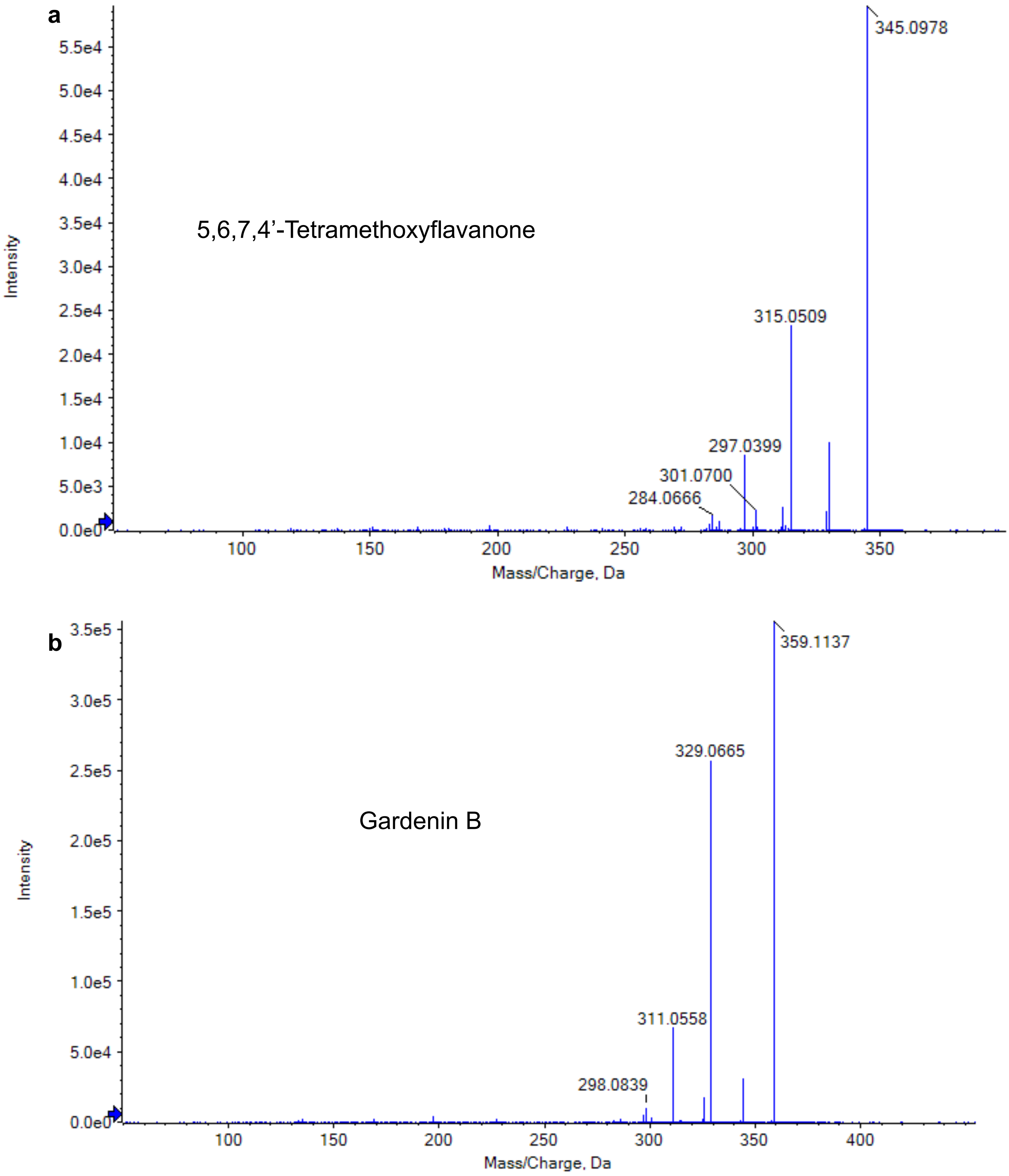

Supplementary Figure 8. Tandem mass spectra for a) 5,6,7,4'-Tetramethoxyflavanone and b) Gardenin B.

a

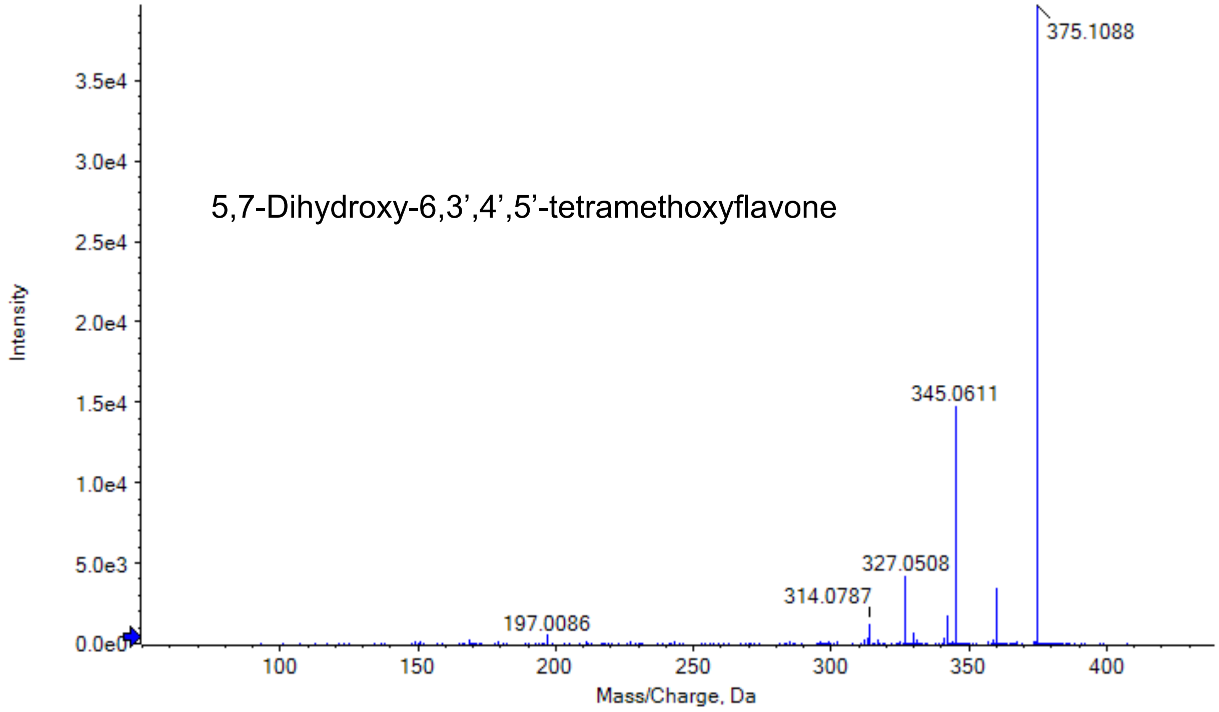

b

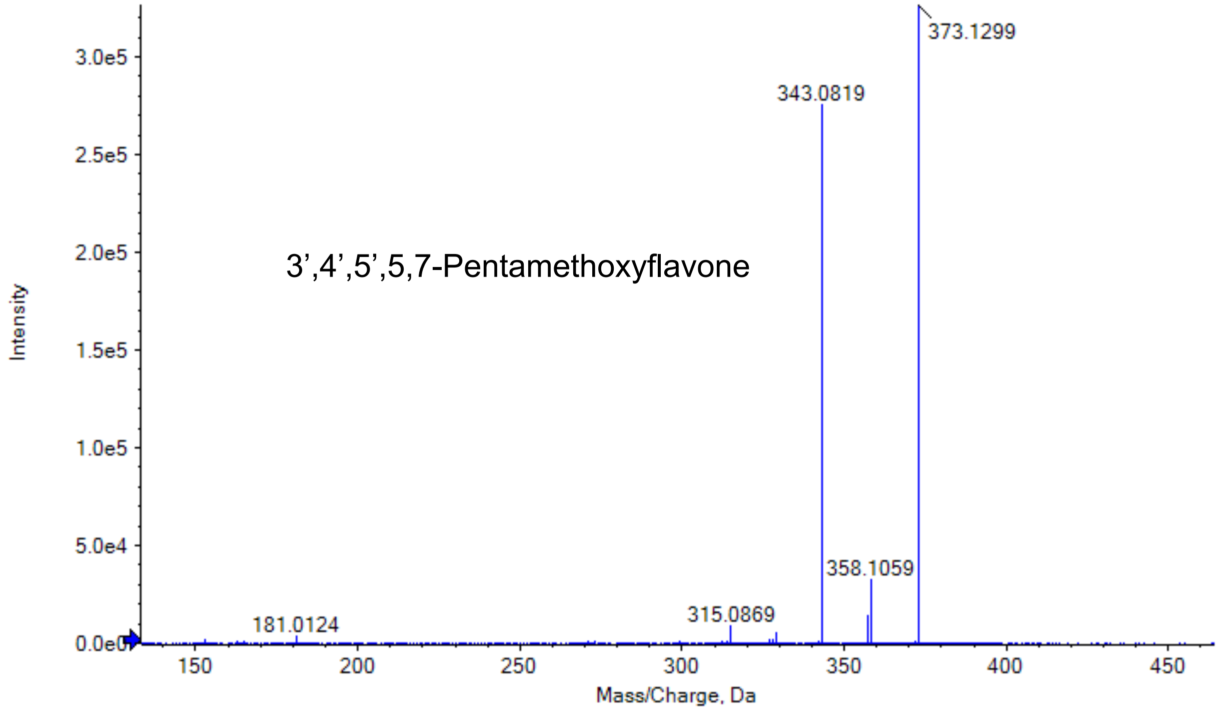

Supplementary Figure 9. Tandem mass spectra for a) 5,7-Dihydroxy-6,3',4',5'-tetramethoxyflavone and b) 3',4',5',5,7-Pentamethoxyflavone.

a

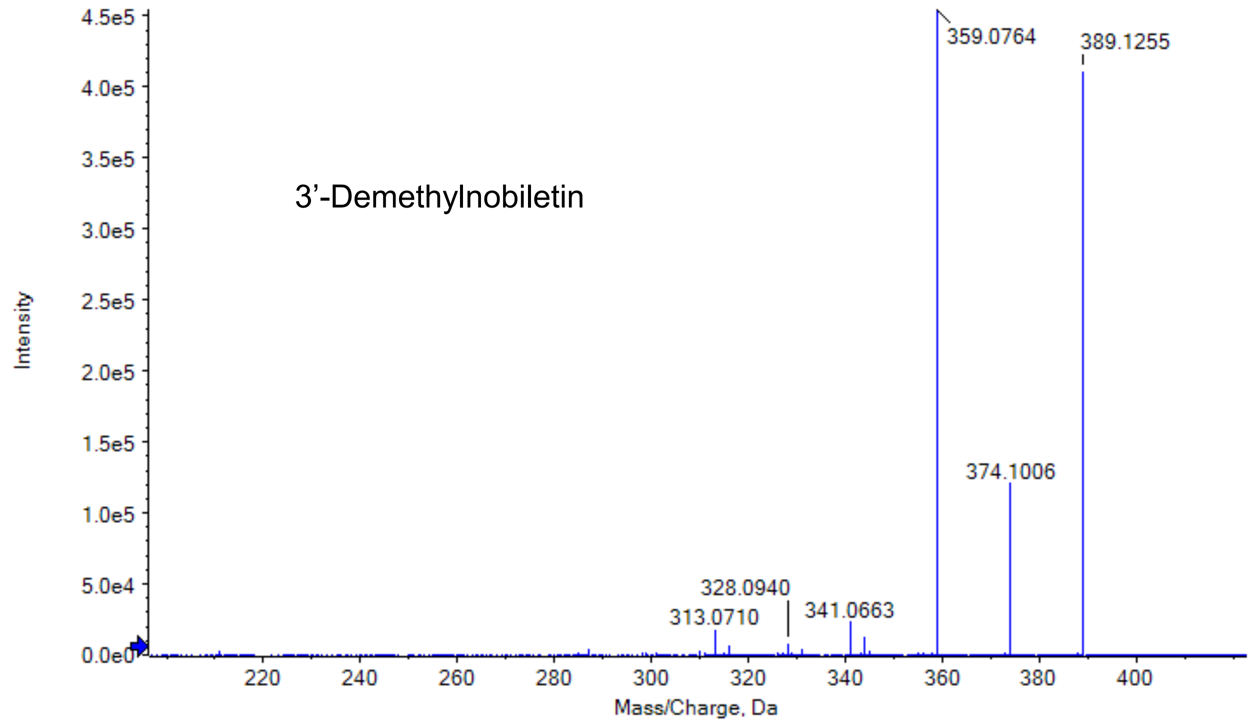

b

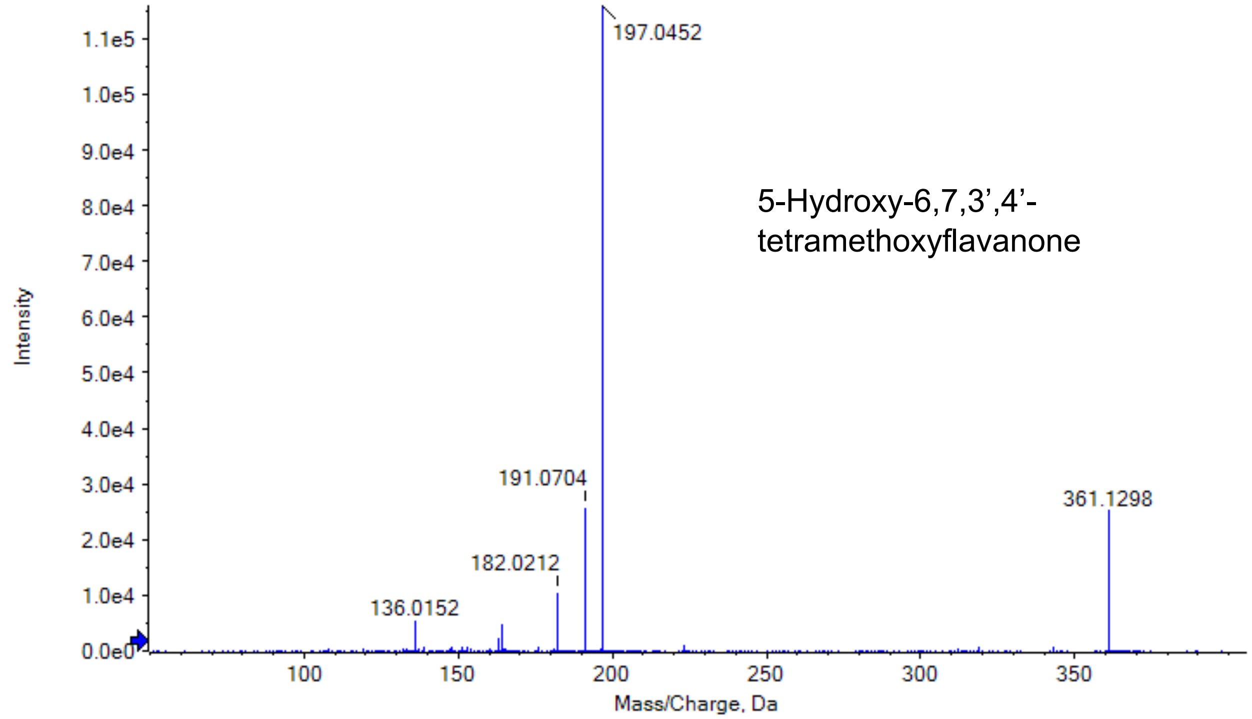

Supplementary Figure 10. Tandem mass spectra for a) 3’-Demethylnobiletin and b) 5-Hydroxy-6,7,3’,4’-tetramethoxyflavanone.

a

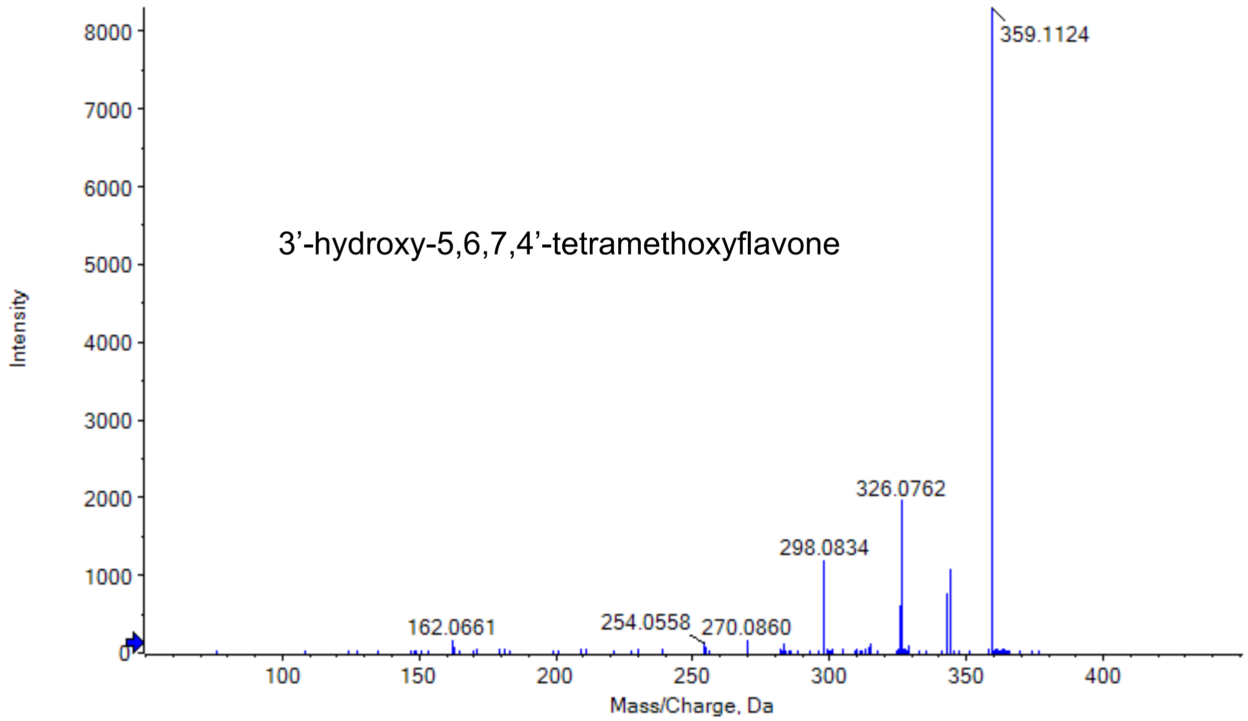

b

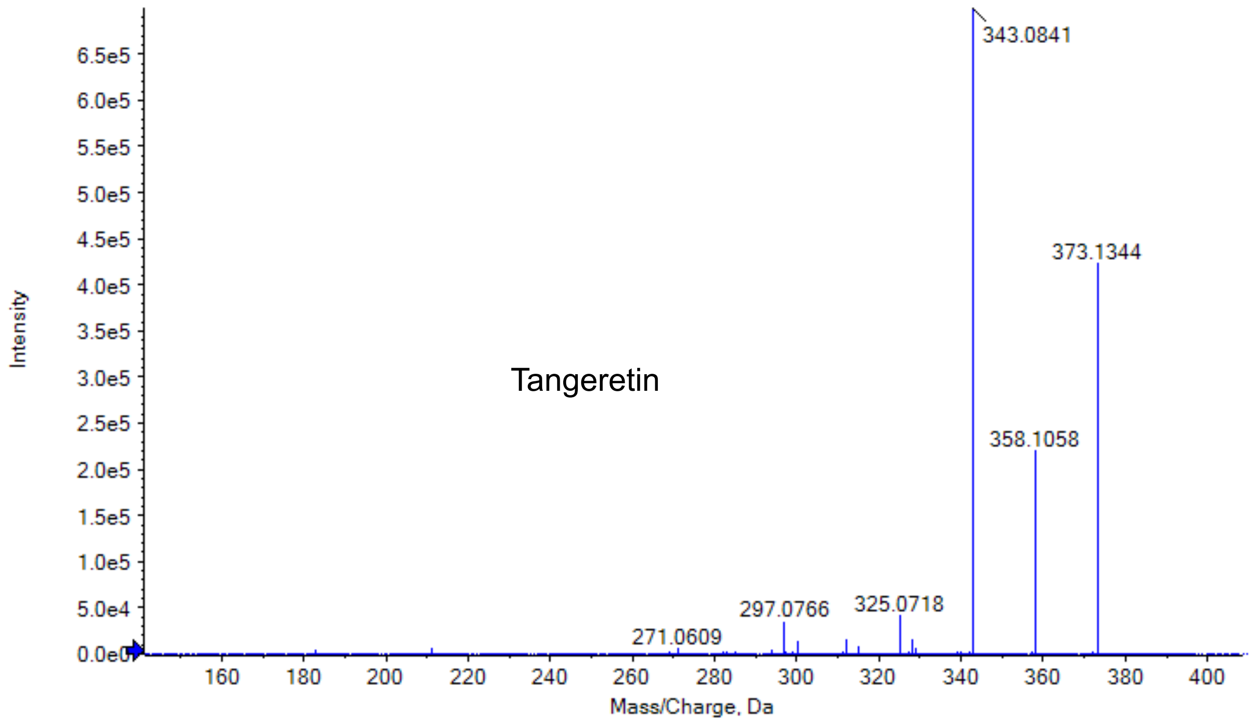

Supplementary Figure 11. Tandem mass spectra for a) 3'-hydroxy-5,6,7,4'-tetramethoxyflavone and b) Tangeretin.

a

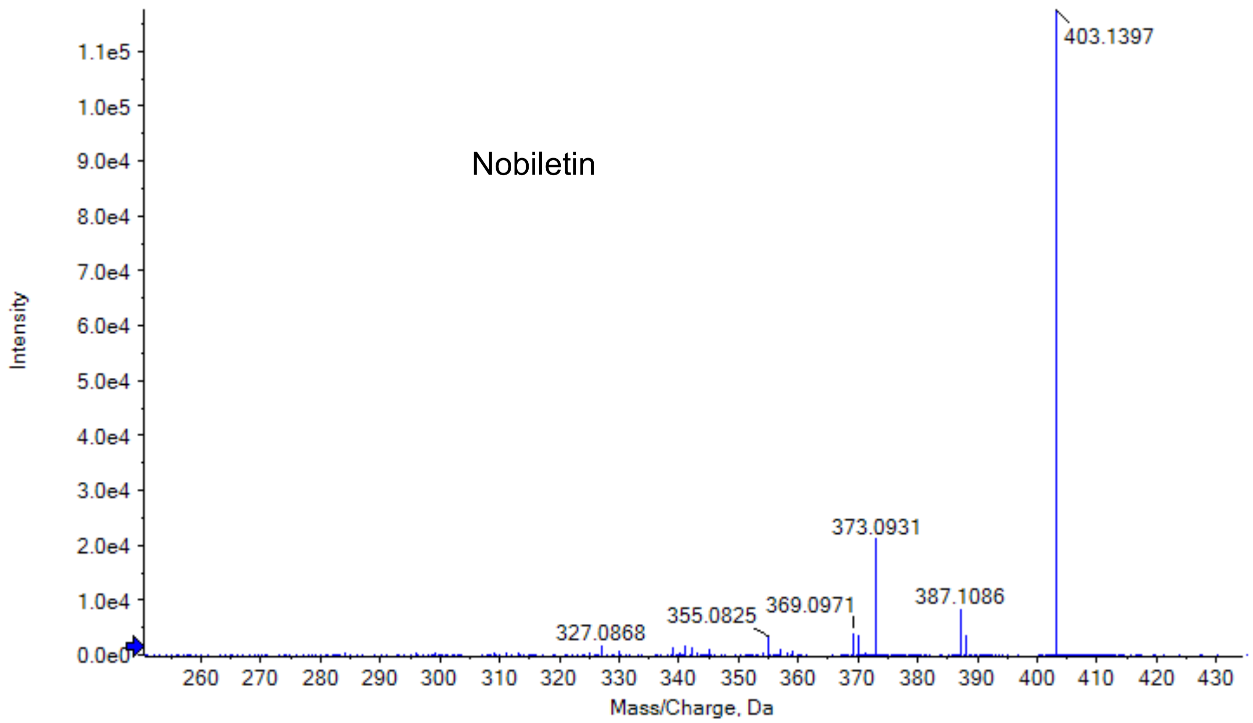

b

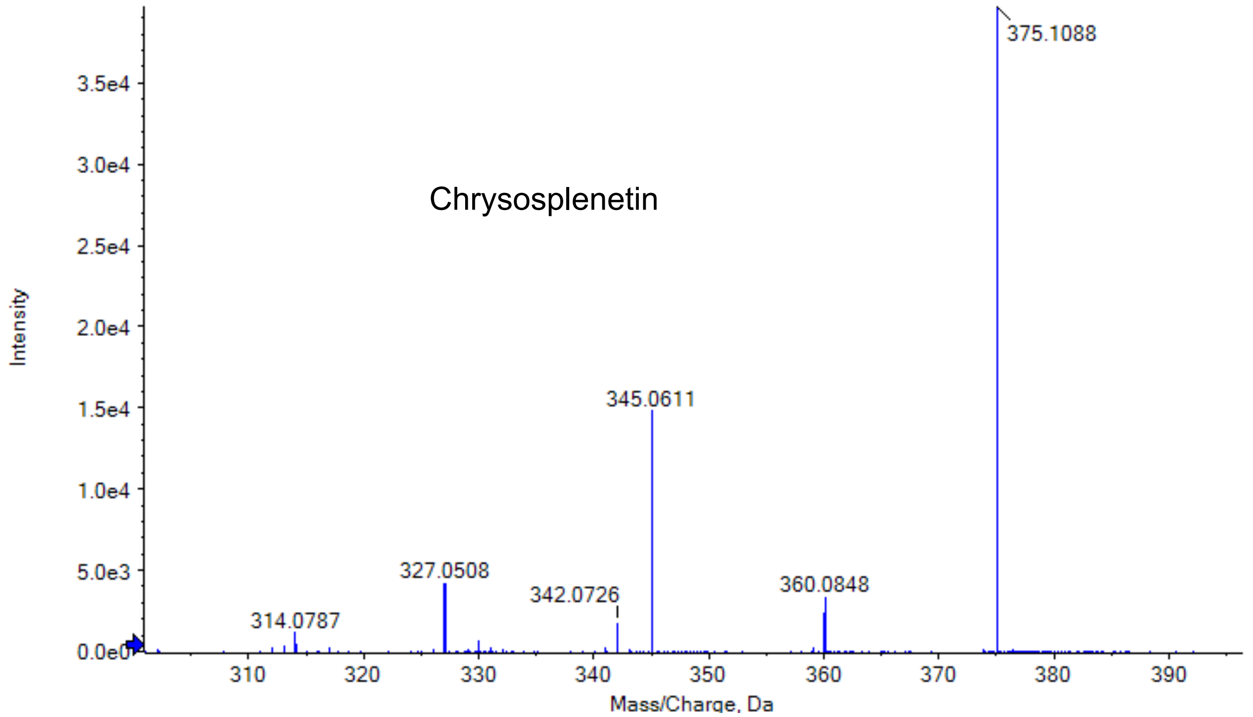

Supplementary Figure 12. Tandem mass spectra for a) Nobiletin and b) Chrysosplenetin.

a

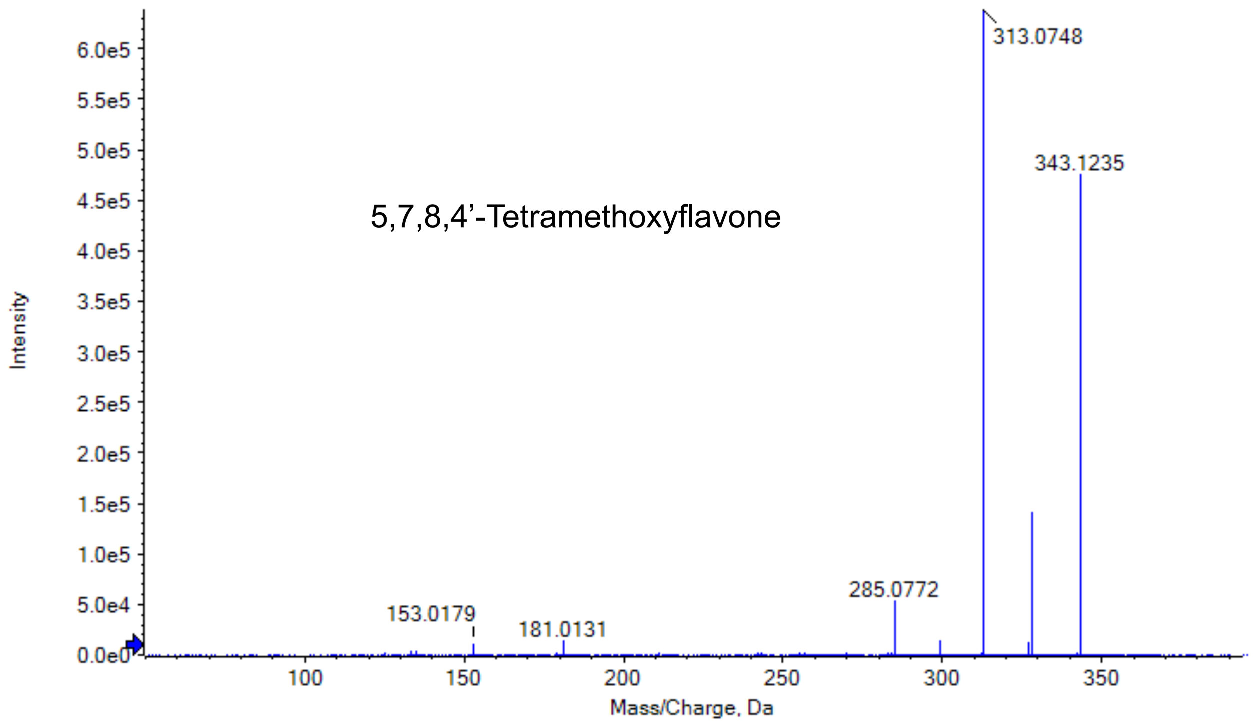

b

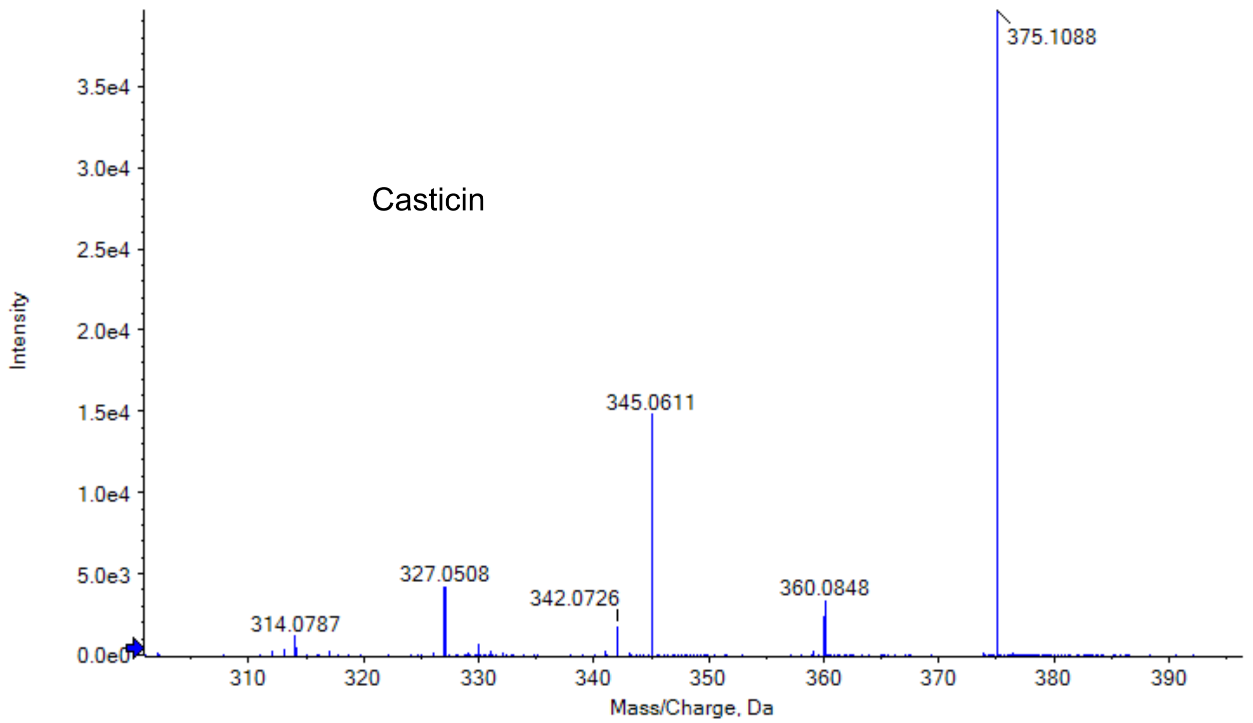

Supplementary Figure 13. Tandem mass spectra for a) 5,7,8,4'-Tetramethoxyflavone and b) Casticin.

a

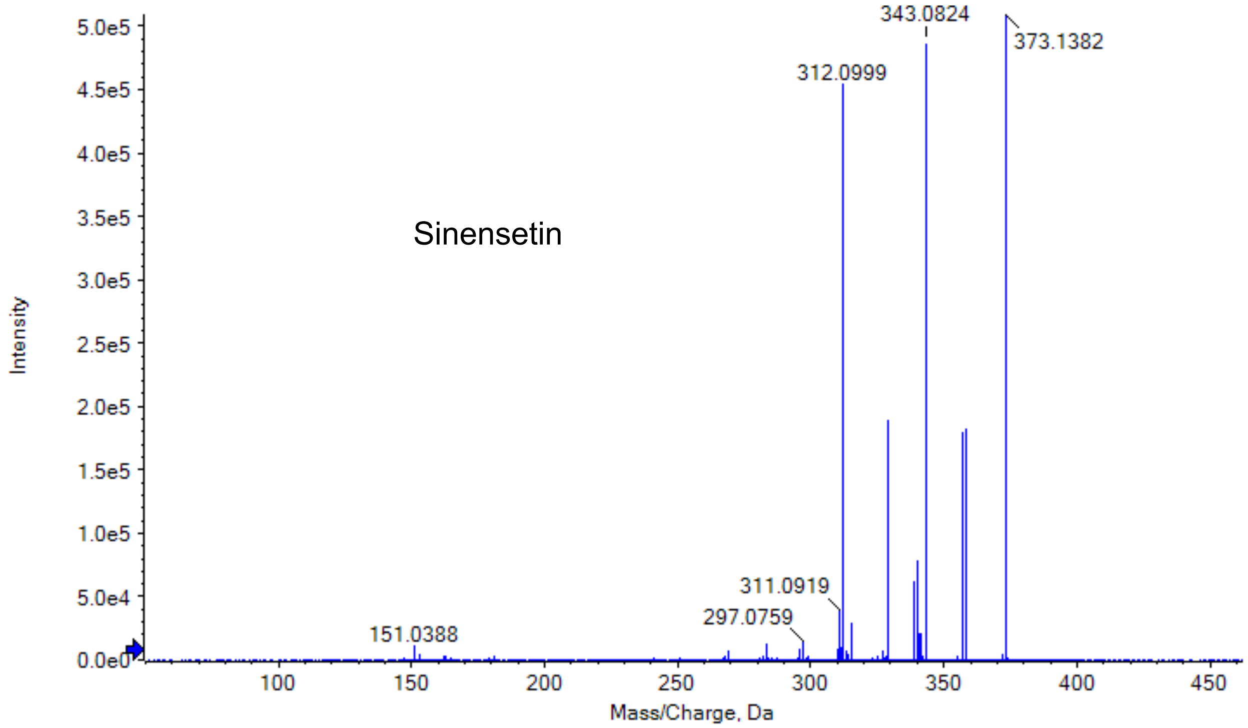

b

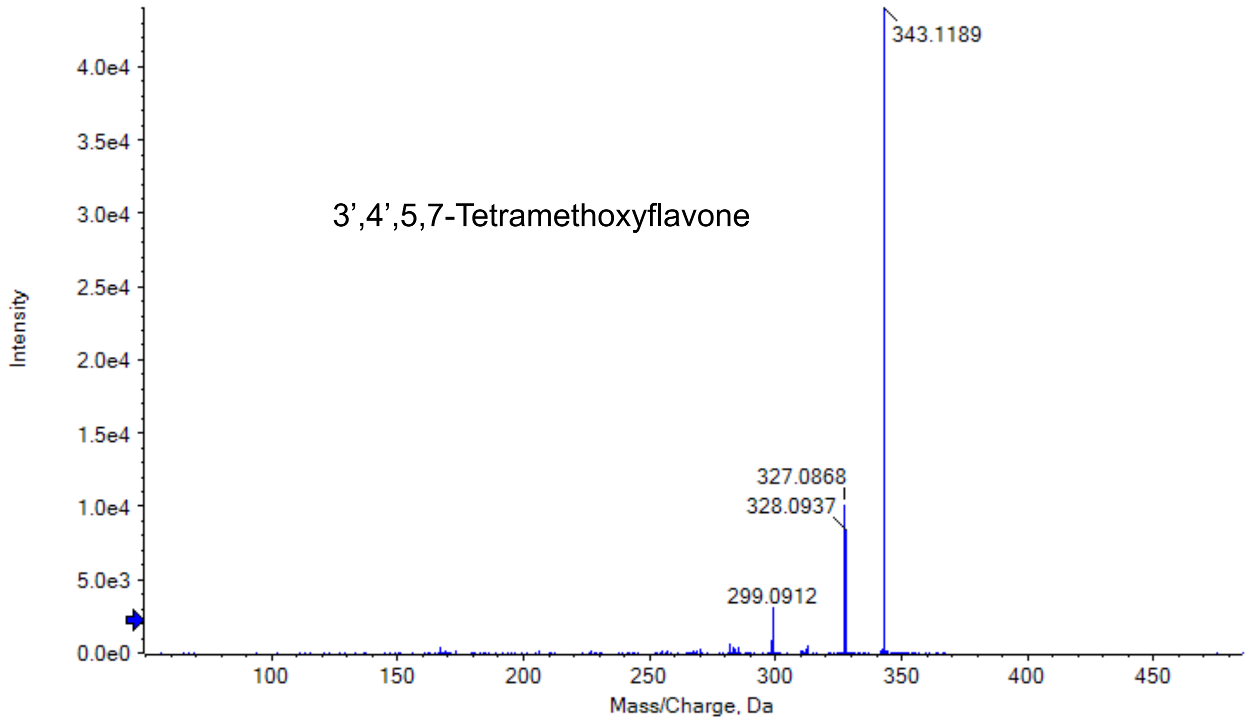

Supplementary Figure 14. Tandem mass spectra for a) Sinensetin and b) 3’,4’,5,7-Tetramethoxyflavone.

a

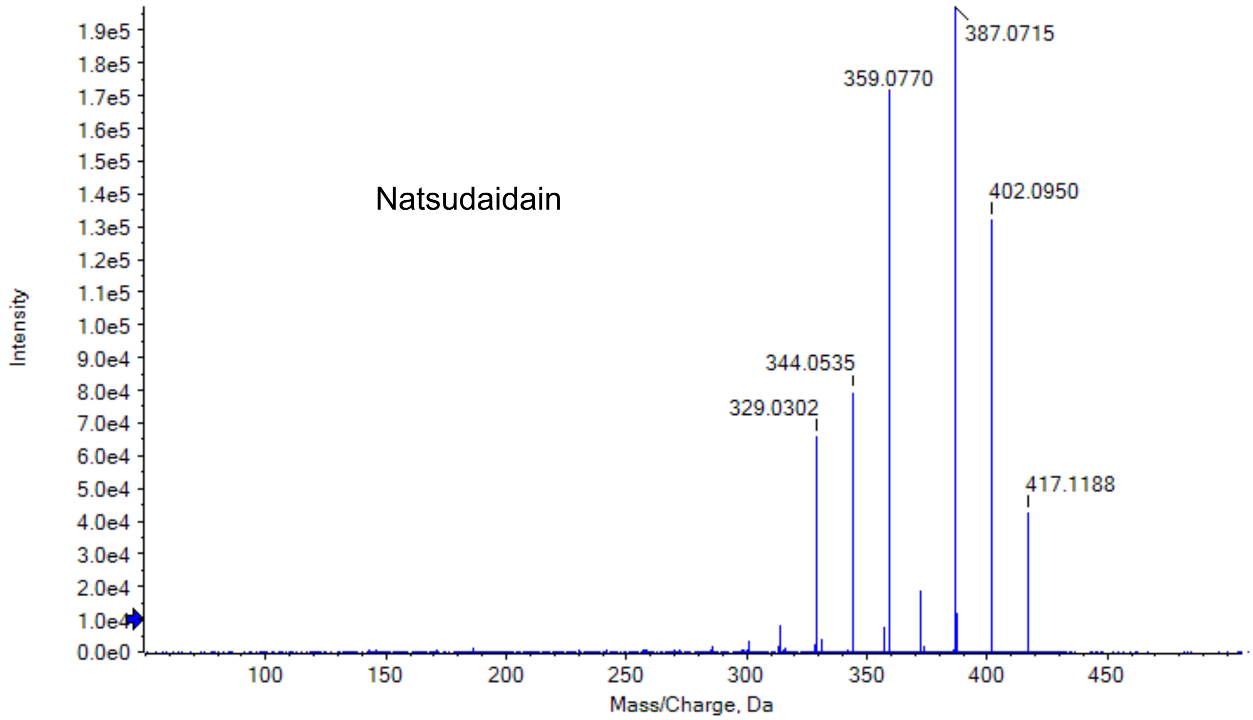

b

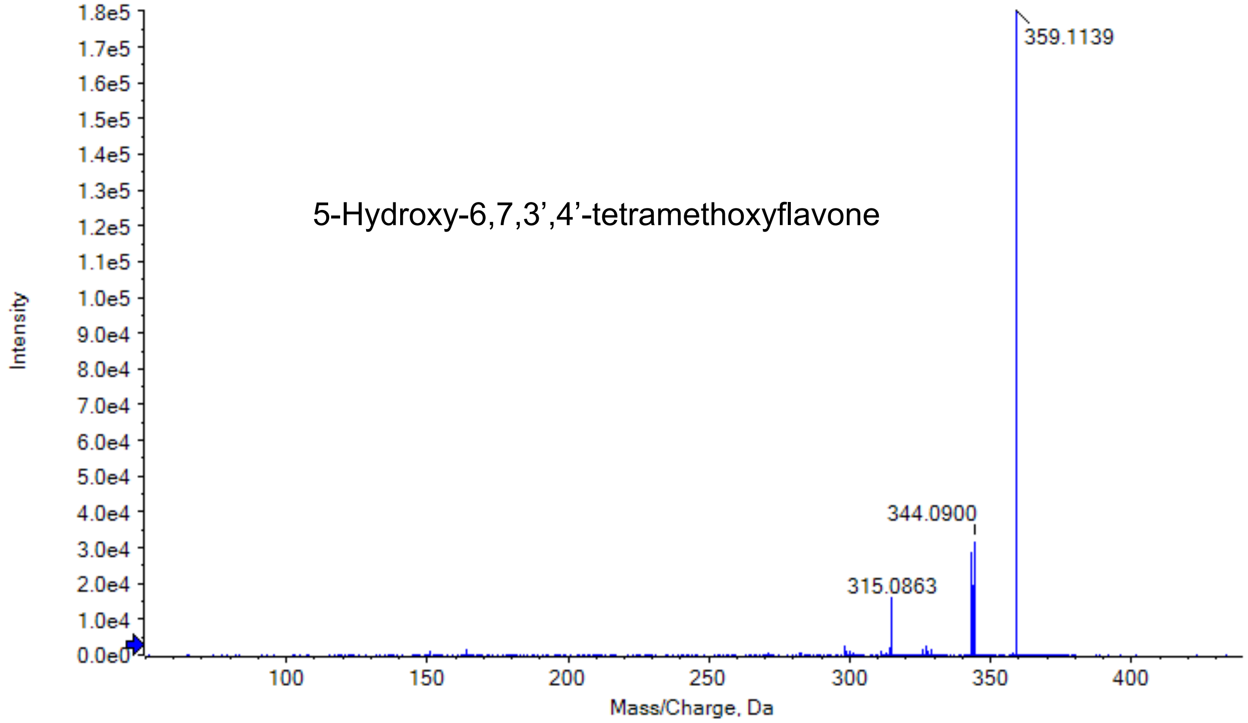

Supplementary Figure 15. Tandem mass spectra for a) Natsudaidain and b) 5-Hydroxy-6,7,3',4'-tetramethoxyflavone.

a

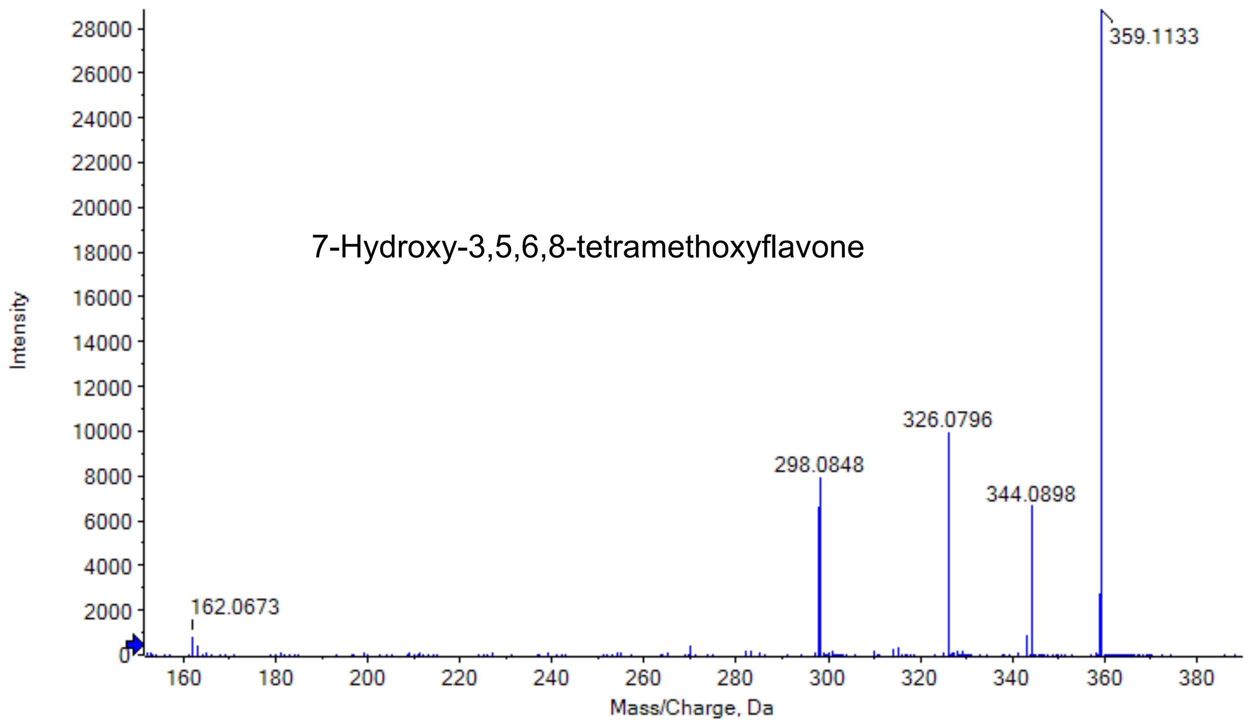

b

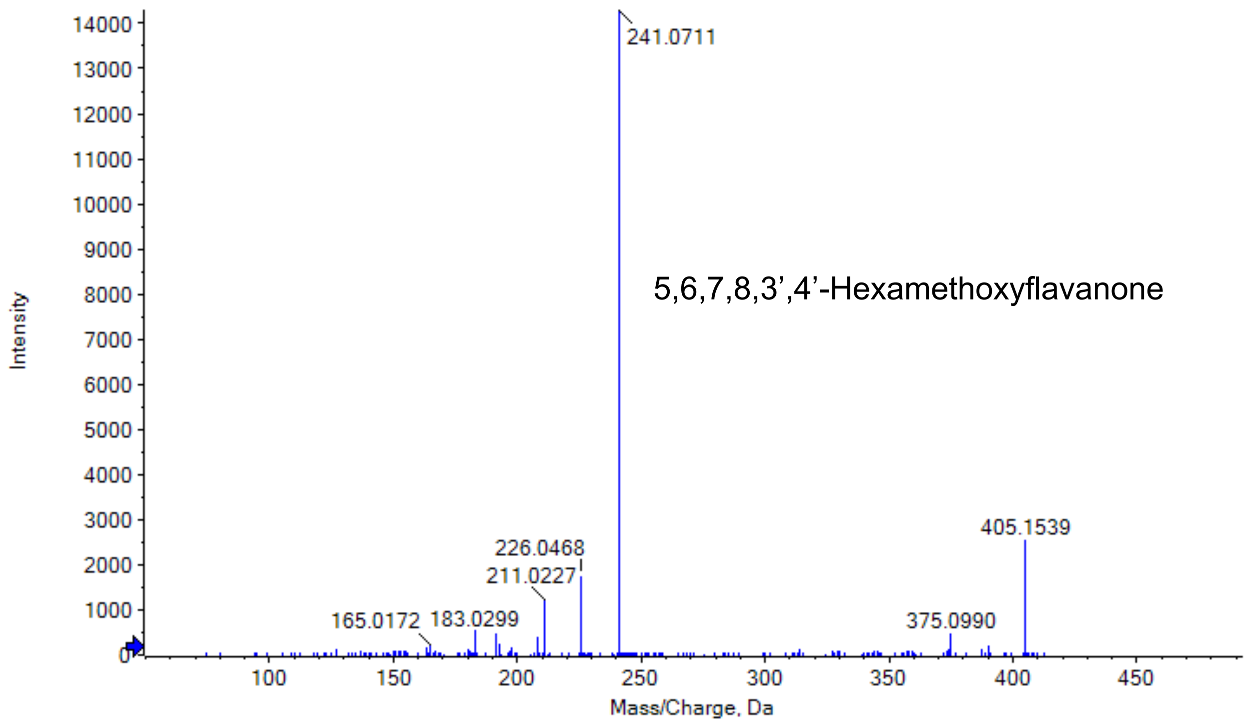

Supplementary Figure 16. Tandem mass spectra for a) 7-Hydroxy-3,5,6,8-tetramethoxyflavone and b) 5,6,7,8,3’,4’-Hexamethoxyflavanone.

a

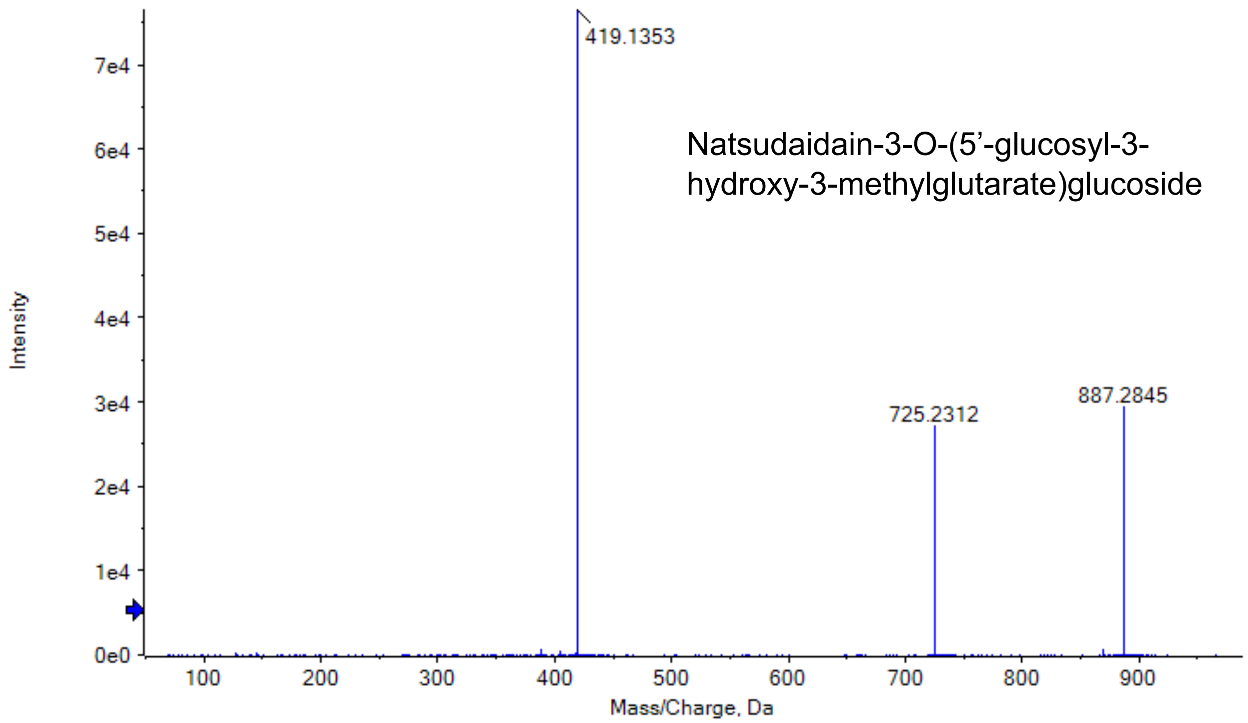

b

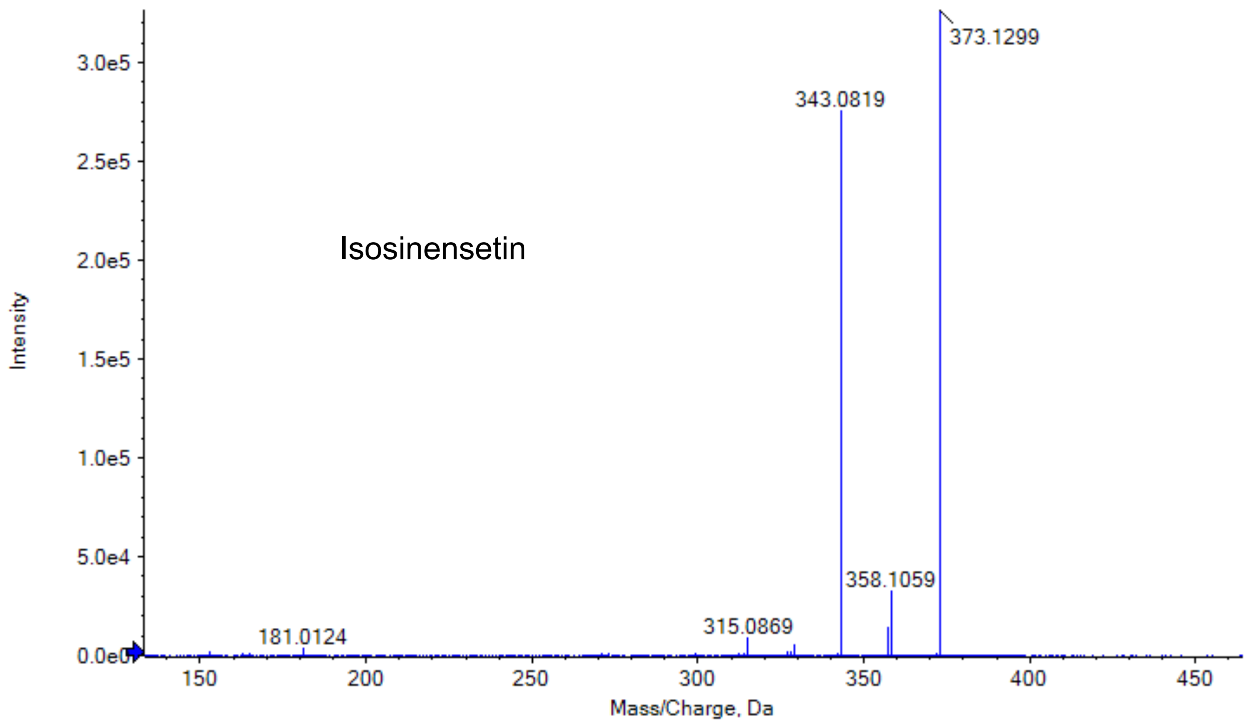

Supplementary Figure 17. Tandem mass spectra for a) Natsudaidain-3-O-(5’-glucosyl-3-hydroxy-3-methylglutarate)glucoside and b) Isosinensetin.

a

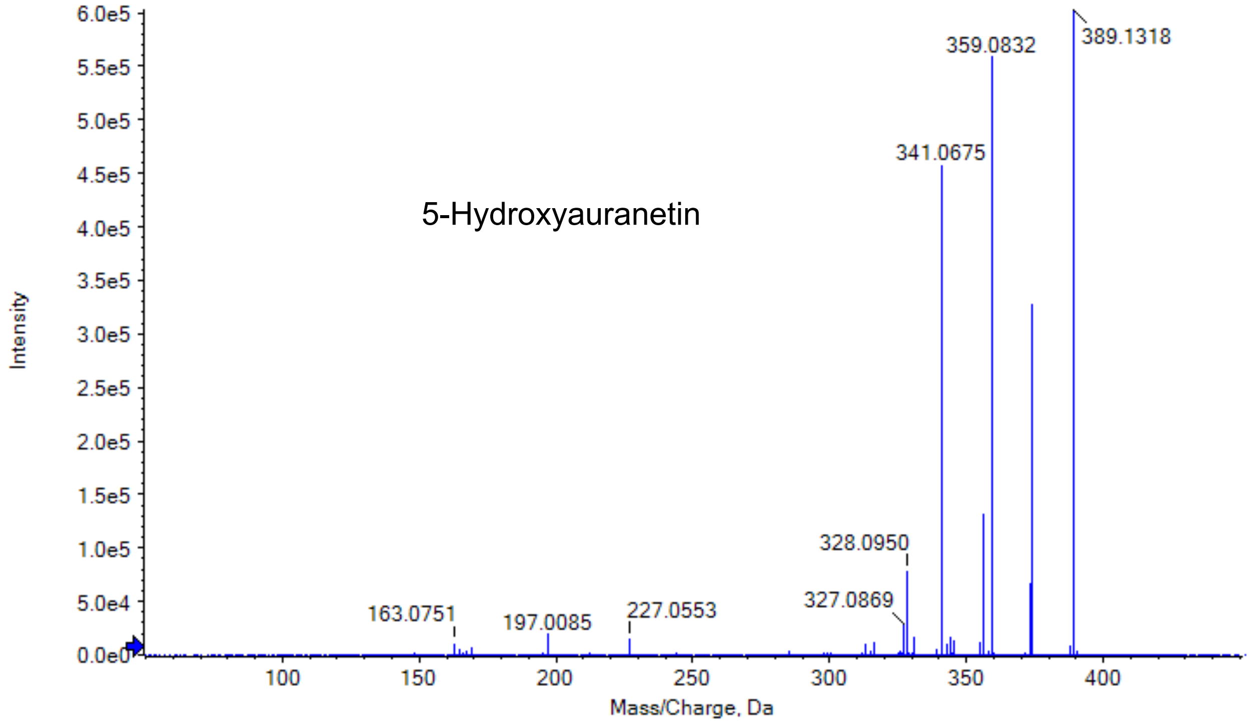

b

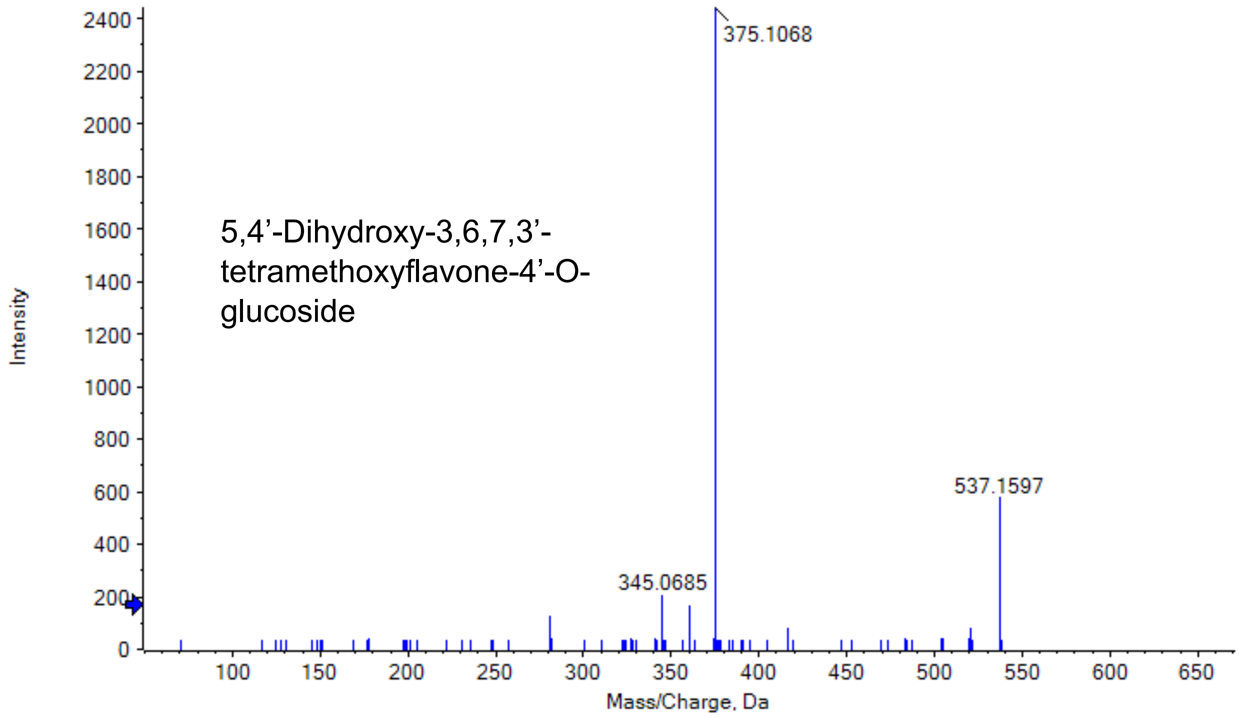

Supplementary Figure 18. Tandem mass spectra for a) 5-Hydroxyauranetin and b) 5,4'-Dihydroxy-3,6,7,3'-tetramethoxyflavone-4'-O-glucoside.

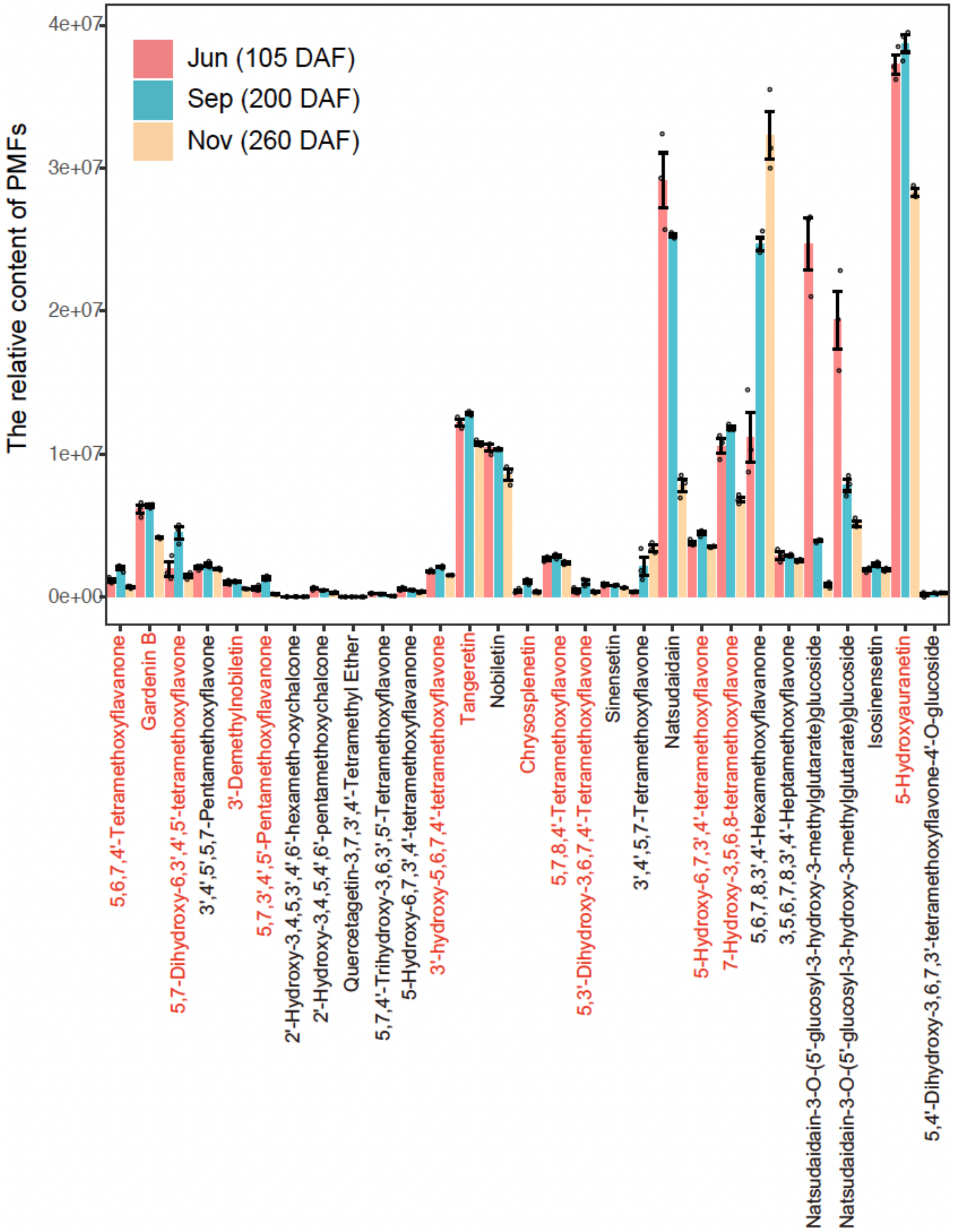

Supplementary Figure 19. The relative content of PMFs in CRC peel at three different stages. Compound name in red indicate the PMF reached its highest relative content at 200 DAF. The relative content of 23 PMFs differ from different stages (one-way ANOVA, $P < 0.05$), 13 of these PMFs show highest relative content at 200 DAF, and the relative content of the 7 PMFs at 200 DAF significant different from 105 DAF and 260 DAF, respectively (Tukey's HSD test, adjusted $P < 0.05$). Error bars, mean $\pm$ s.d., n=3. Source data are provided.

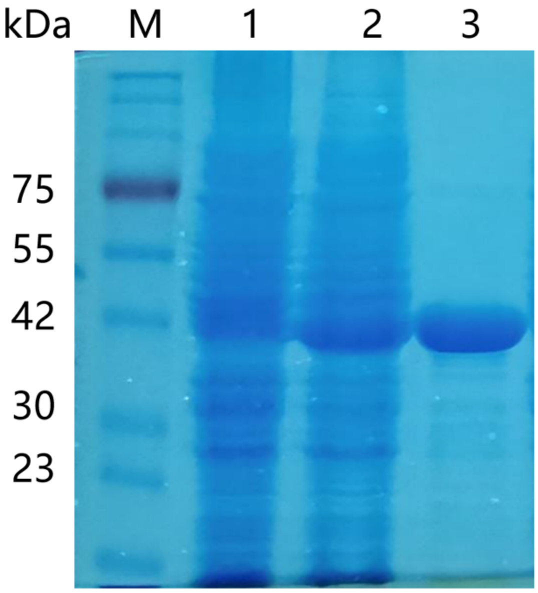

Supplementary Figure 20. SDS-PAGE analysis of recombinant CcOMT1 proteins. Line 1: uninduced; Line 2: supernatant of induced bacterial cells; Line 3: purified recombinant proteins. This experiment was repeated 3 times with similar results. Source data are provided.

a

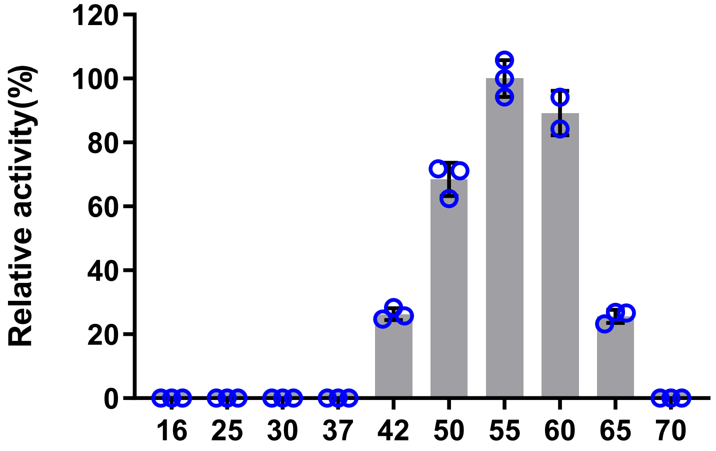

b

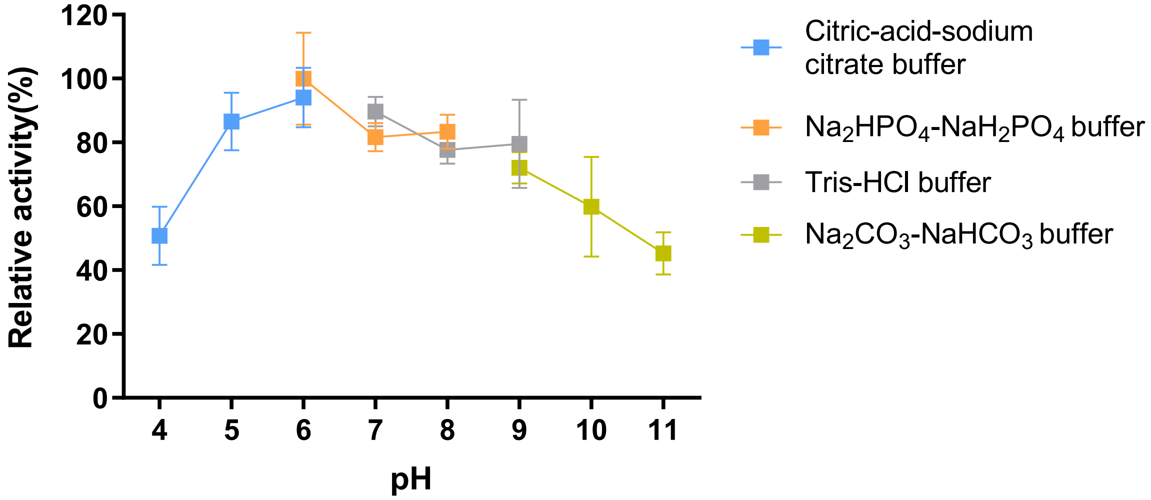

Supplementary Figure 21. Effects of temperature (a) and pH (b) on the enzyme activity of CcOMT1. SAM was used as the methyl donor and natsudaidain was used as the acceptor. CcOMT1 showed the optimal reaction temperatures at 55°C, and the maximum activity at pH 6.0. Error bars, mean $\pm$ s.d., n = 3 for all the data except 60°C (n=2 for 60°C). Source data are provided.

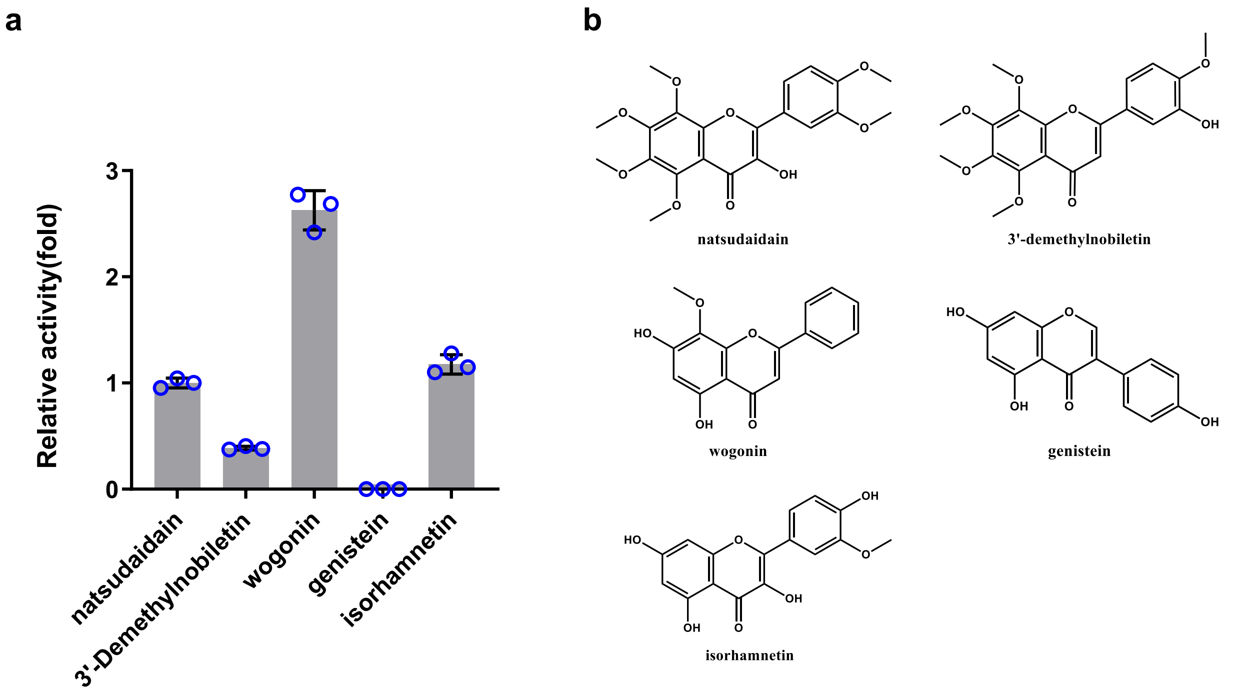

Supplementary Figure 22. Substrate promiscuity of CcOMT1. a The yields of methylated products catalyzed by CcOMT1. **b** Structures of substrates for CcOMT1. Error bars, mean ± s.d., n=3. Source data are provided.

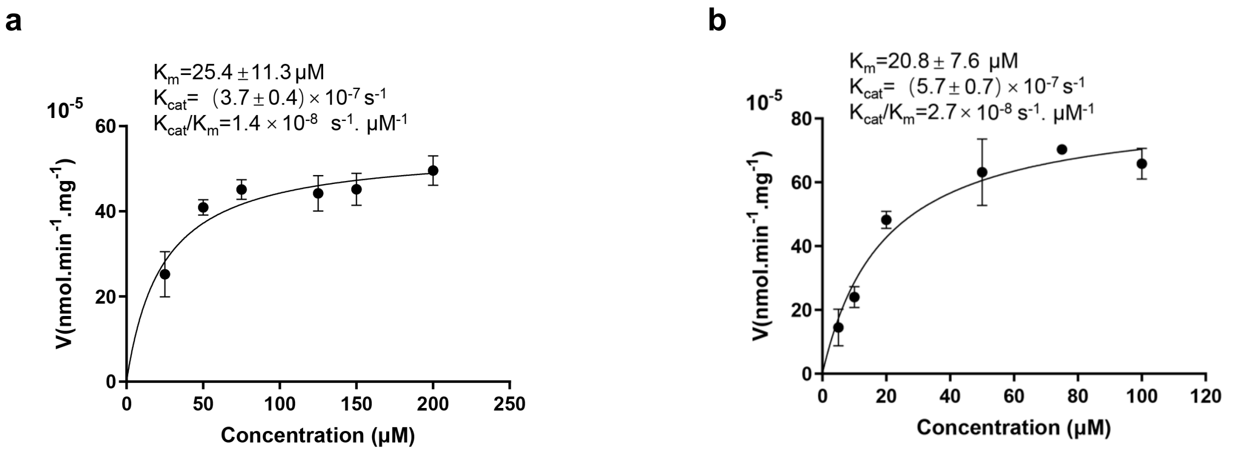

Supplementary Figure 23. Determination of kinetic parameters for CcOMT1 (a) and the mutant CcOMT1-W141Y (b). The Km value of CcOMT1 and CcOMT1-W141Y for natsudaidain (**1**) are 25.4±11.3 μM and 20.8±7.6 μM, respectively. The Kcat/Km value of CcOMT1 and CcOMT1-W141Y on natsudaidain (**1**) are 1.4× 10^{-8} s^{-1}. μM^{-1} and 2.7× 10^{-8} s^{-1}. μM^{-1}, respectively. Source data are provided.

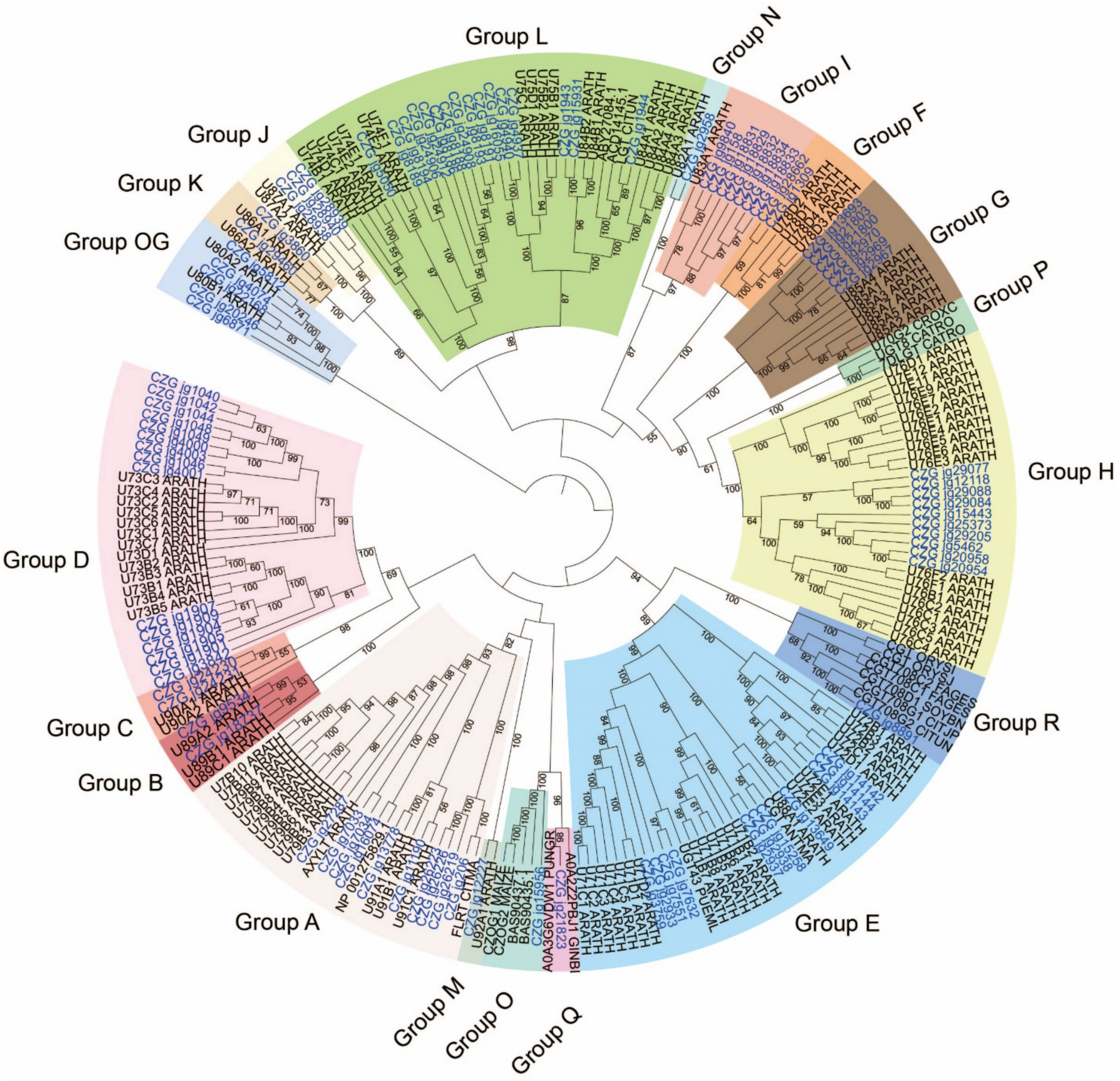

Supplementary Figure 24. Maximum likelihood tree of UGT genes of CRC and other plants.

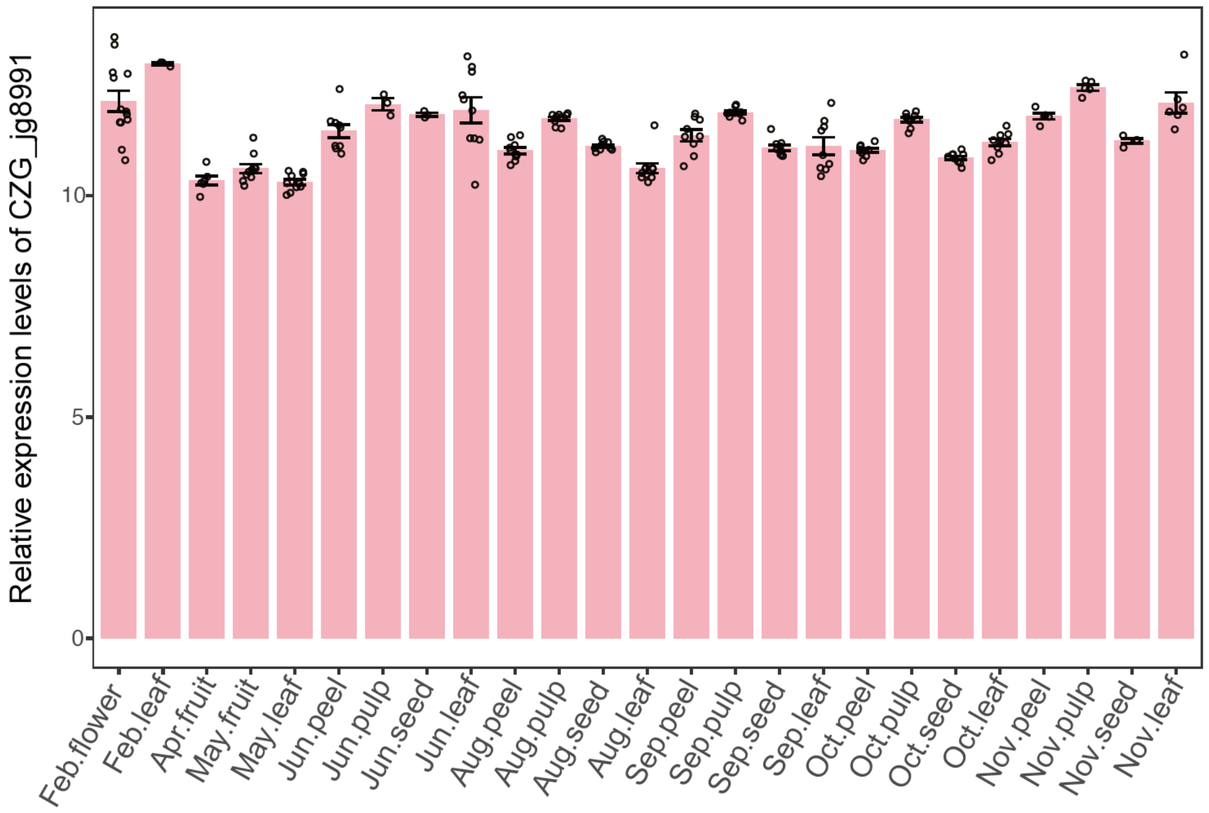

Supplementary Figure 25. The relative expression levels of the candidate CGT gene CZG_jg8991. Error bars, mean ± s.d. For flowers samples, n=13; for samples of May fruit, May leaf, Jun leaf, Aug leaf and Aug peel, n=10, respectively; for samples of Jun peel, Aug seed, Aug pulp, Sep leaf, Sep peel, Sep pulp, Sep seed, Oct pulp,Oct leaf, Oct peel, and Oct seed, n=9, respectively; for samples of Apr fruit and Nov leaf, n =6, respectively; for samples of Nov peel and Nov pulp, n=4, respectively; for samples of Nov seed, n=4; for samples of Feb leaf, Jun pulp and Jun seed, n=3, respectively. Source data are provided.

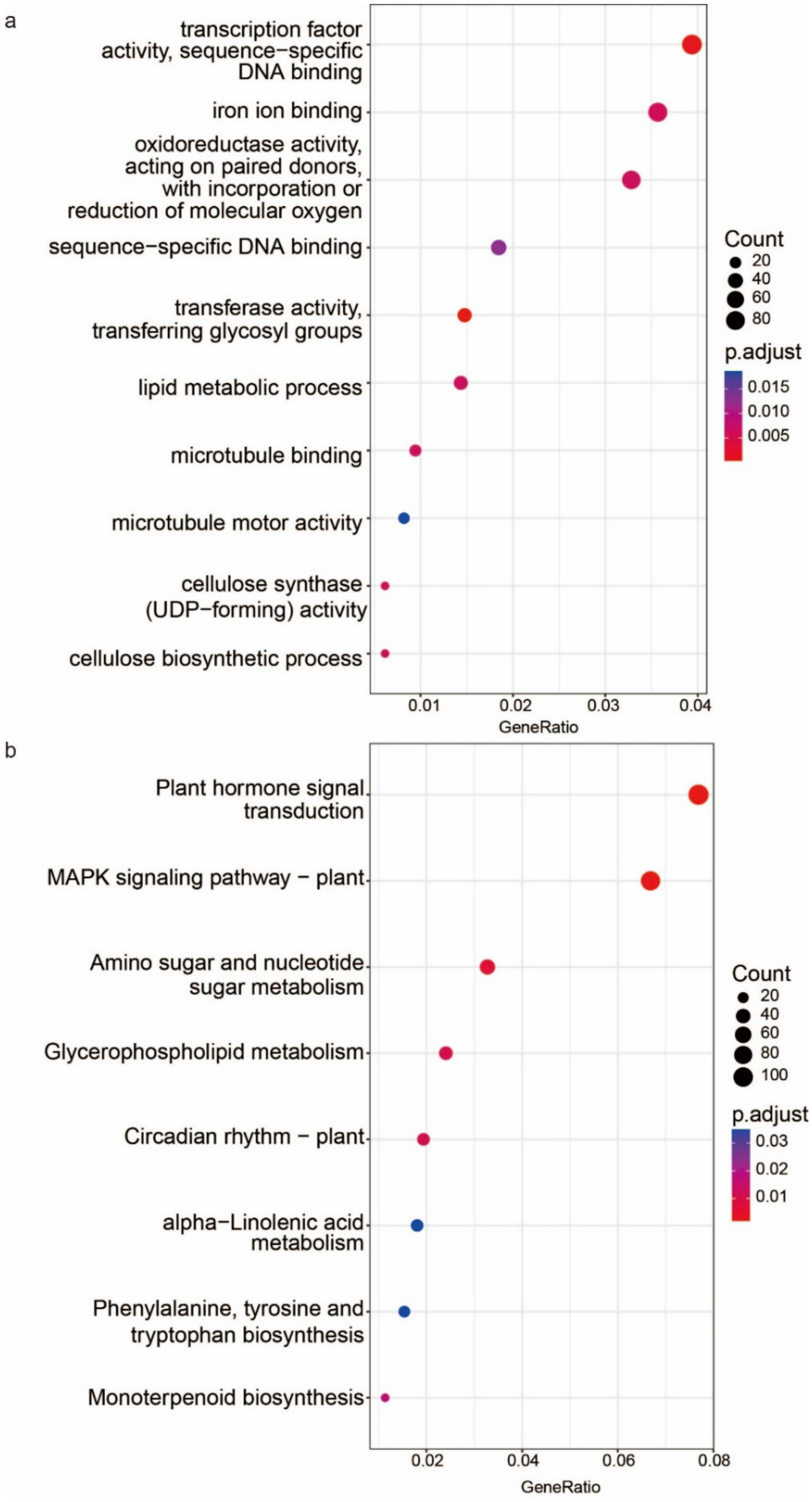

Supplementary Figure 26. Functional enrichment analysis of differentially expressed genes of peel between 105 DAF and 200 DAF at CRC. a GO enrichment analysis result. **b** KEGG enrichment analysis result.

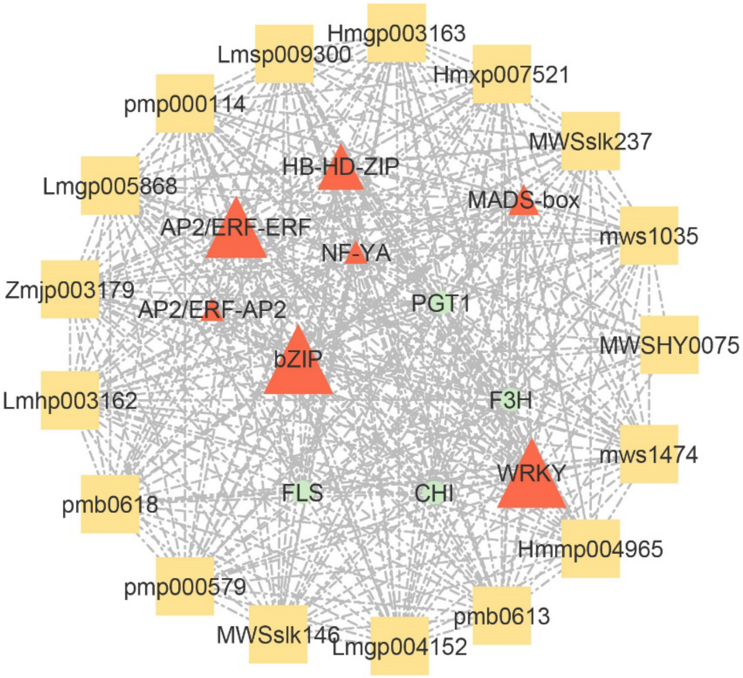

Supplementary Figure 27. The correlation network of DEGs and DMs between 105 DAF and 200 DAF. Squares indicate differentially accumulated metabolites (DMs); triangles indicate differentially expressed transcription factors; green circles indicate differentially expressed flavonoid biosynthesis genes; gray dash represent the positive correlation (r > 0.8, $P < 0.05$).

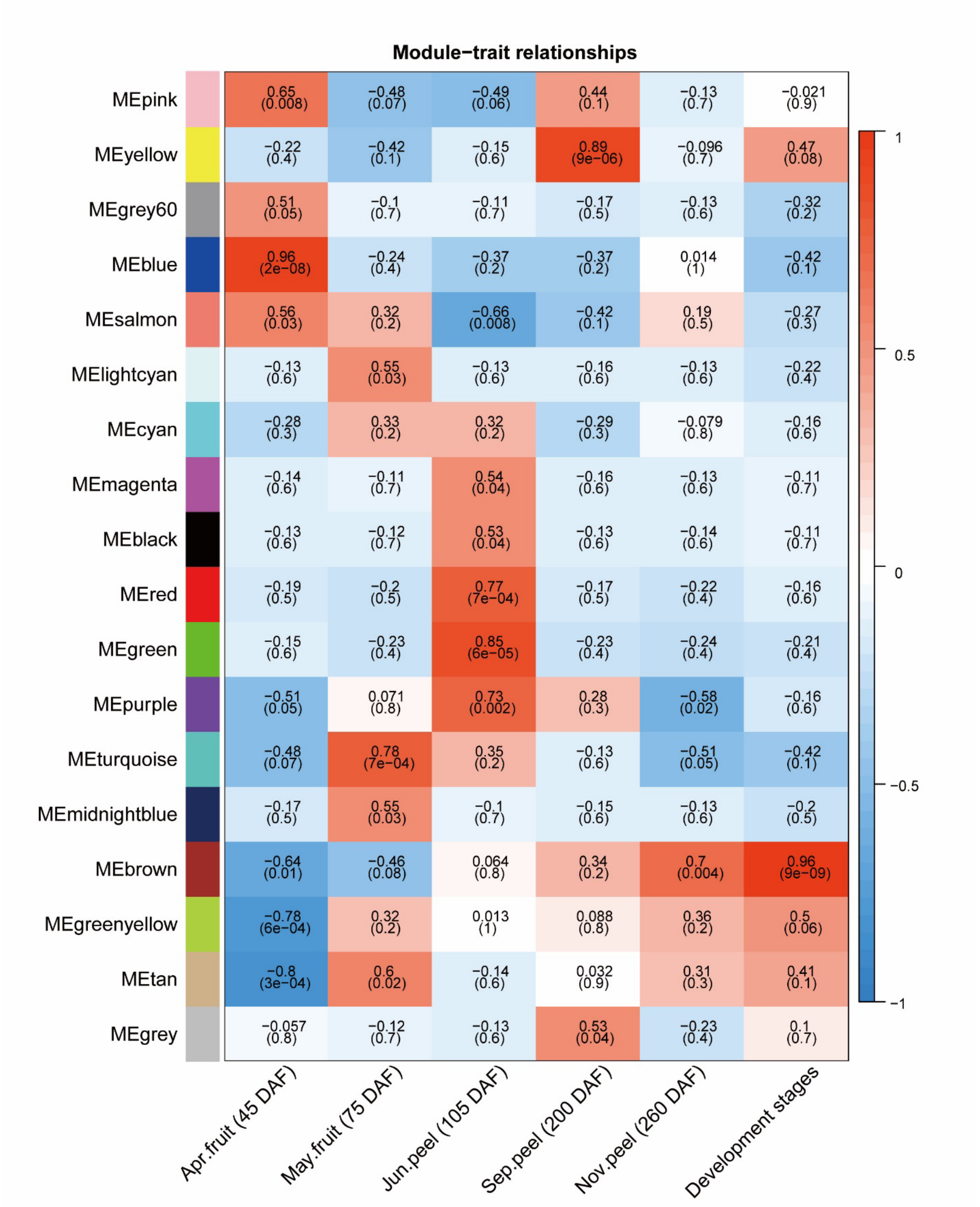

Supplementary Figure 28. The relationship between modules and fruit developmental stages.

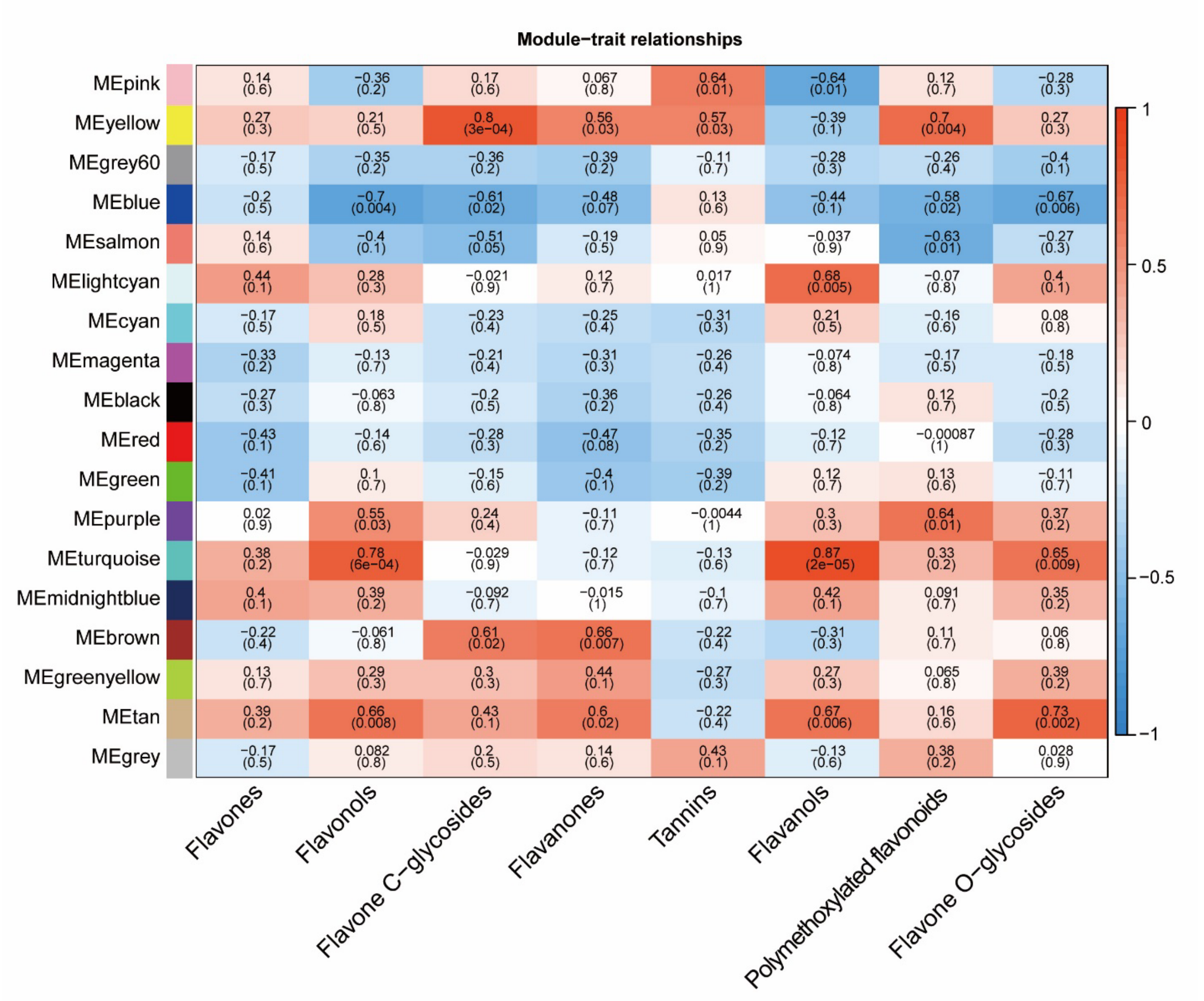

Supplementary Figure 29. The relationship between modules and the relative content of different kinds of flavonoids.
