# Supplementary material for: An integrated multi-omics approach reveals polymethoxylated flavonoid biosynthesis in Citrus reticulata cv. Chachiensis
Source: Nat Commun. 2024 May 11;15:3991. doi: 10.1038/s41467-024-48235-y (PMC11088696; doi:10.1038/s41467-024-48235-y)
Supplement: Supplementary file 3 — Description of Additional Supplementary Files [file 41467_2024_48235_MOESM3_ESM.pdf]

Description of Additional Supplementary Files:

Supplementary Data 1: Summary of 9 chromosome-scale pseudomolecules.

Supplementary Data 2: Repetitive sequence annotation of CRC genome.

Supplementary Data 3: The summary of functional annotation results.

Supplementary Data 4: The summary of transcription factor annotation results.

Supplementary Data 5: The summary of non-coding RNA annotation results.

Supplementary Data 6: Functional enrichment of expanded gene families.

Supplementary Data 7: The detected flavonoids in CRC.

Supplementary Data 8: The relative content of PMFs in fruits and peels of CRC.

Supplementary Data 9: Differentially accumulated metabolites between 45 DAF and 75 DAF.

Supplementary Data 10: Differentially accumulated PMFs in citrus peels at 105 DAF, 200 DAF and 260 DAF.

Supplementary Data 11: The function of flavonoid-related OMTs.

Supplementary Data 12: The genes and metabolites in the correlation network.

Supplementary Data 13: The correlation between AP2/ERF-ERF and 5,6,7,8,3’,4’-hexamethoxyflavone.

Supplementary Data 14: Primers for site-directed mutagenesis of CcOMT1.

Supplementary Data 15: Primers used for cloning CcOMT1 and RT–qPCR.
