# Supplementary material for: An integrated multi-omics approach reveals polymethoxylated flavonoid biosynthesis in Citrus reticulata cv. Chachiensis
Source: Nat Commun. 2024 May 11;15:3991. doi: 10.1038/s41467-024-48235-y (PMC11088696; doi:10.1038/s41467-024-48235-y)
Supplement: Supplementary file 19 — Reporting Summary [file 41467_2024_48235_MOESM19_ESM.pdf]

nature portfolio

Corresponding author(s): Huan Liu (liuhuan@genomics.cn)

Last updated by author(s): 04/07/2024

Reporting Summary

Nature Portfolio wishes to improve the reproducibility of the work that we publish. This form provides structure for consistency and transparency in reporting. For further information on Nature Portfolio policies, see our Editorial Policies and the Editorial Policy Checklist.

Statistics

For all statistical analyses, confirm that the following items are present in the figure legend, table legend, main text, or Methods section.

n/a	Confirmed	
☐	☒	The exact sample size (n) for each experimental group/condition, given as a discrete number and unit of measurement
☐	☒	A statement on whether measurements were taken from distinct samples or whether the same sample was measured repeatedly
☐	☒	The statistical test(s) used AND whether they are one- or two-sided<br>*Only common tests should be described solely by name; describe more complex techniques in the Methods section.*
☒	☐	A description of all covariates tested
☒	☐	A description of any assumptions or corrections, such as tests of normality and adjustment for multiple comparisons
☐	☒	A full description of the statistical parameters including central tendency (e.g. means) or other basic estimates (e.g. regression coefficient) AND variation (e.g. standard deviation) or associated estimates of uncertainty (e.g. confidence intervals)
☐	☒	For null hypothesis testing, the test statistic (e.g. F, t, r) with confidence intervals, effect sizes, degrees of freedom and P value noted<br>*Give P values as exact values whenever suitable.*
☐	☒	For Bayesian analysis, information on the choice of priors and Markov chain Monte Carlo settings
☒	☐	For hierarchical and complex designs, identification of the appropriate level for tests and full reporting of outcomes
☐	☒	Estimates of effect sizes (e.g. Cohen's d, Pearson's r), indicating how they were calculated

Our web collection on statistics for biologists contains articles on many of the points above.

Software and code

Policy information about availability of computer code

Data collection

No software was used for data collection.

Data analysis

The list of software used in this study show below:
GenomeScope (v1.0),
Nextdenovo (v2.2 beta.0),
Nextpolish (v1.3.0),
Purge_dups (v1.2.5),
3D-DNA (v180922),
BUSCO (v3.01, embryophyta_odb10, 1375),
PILER (v1.0),
RepeatScout (v1.0.5),
LTR_FINDER (v1.06),
RepeatClassifier (v1.08),
RepeatMasker (open-4.0.6),
Repeats Finder (v4.07b),
BRAKER2 (v2.1.5),
TopHat2 (version 2.1.0),
iTak (v1.4),
tRNAscan-SE (v1.3.1),

BLAST (v2.2.26),
OrthoFinder (v2.5.2),
MAFFT (v.7.310),
RAxML (v8.2.12),
PAML package,
CAFE5 (v1.1),
MCscan (Python version),
wgd (v1.1.0),
DupGen_finder,
STAR (v2.7.9a),
featureCounts (Subread v2.0.1),
R package DESeq2,
R package clusterProfiler 4.0,
WGCNA package (v1.70-3),
Cytoscape (v3.9.0),
R package Hmisc (v4.60),
Robbeta,
Pocasa,
Autodock Vina version 1.5.6

For manuscripts utilizing custom algorithms or software that are central to the research but not yet described in published literature, software must be made available to editors and reviewers. We strongly encourage code deposition in a community repository (e.g. GitHub). See the Nature Portfolio guidelines for submitting code & software for further information.

Data

Policy information about availability of data

All manuscripts must include a data availability statement. This statement should provide the following information, where applicable:

- Accession codes, unique identifiers, or web links for publicly available datasets
- A description of any restrictions on data availability
- For clinical datasets or third party data, please ensure that the statement adheres to our policy

The raw RNA-seq data and genome sequences have been deposited in the Genome Sequence Archive (GSA) in National Genomics Data Center, China National Center for Bioinformation/Beijing Institute of Genomics, Chinese Academy of Sciences under accession number CRA015571 (https://ngdc.cncb.ac.cn/gsa/browse/CRA015571). The chromosome-level genome assembly and the scaffold-level genome assembly data have been deposited in the Genome Warehouse under accession numbers GWHERQK00000000 (https://ngdc.cncb.ac.cn/gwh/Assembly/84113/show) and GWHERPZ00000000 (https://ngdc.cncb.ac.cn/gwh/Assembly/84102/show), respectively. The transcriptome, genome sequence and genome assembly data have also been deposited in CNGBdb under accession codes CNP0003922 (https://db.cngb.org/search/?q=CNP0003922) and CNP0003929 (https://db.cngb.org/search/?q=CNP0003929), respectively. The metabolomics data have been deposited to the EMBL-EBI MetaboLights database with the identifier MTBLS9832 (https://www.ebi.ac.uk/metabolights/MTBLS9832). Source data are provided with this paper.

Research involving human participants, their data, or biological material

Policy information about studies with human participants or human data. See also policy information about sex, gender (identity/presentation), and sexual orientation and race, ethnicity and racism.

Reporting on sex and gender	Not applicable
Reporting on race, ethnicity, or other socially relevant groupings	Not applicable
Population characteristics	Not applicable
Recruitment	Not applicable
Ethics oversight	Not applicable

Note that full information on the approval of the study protocol must also be provided in the manuscript.

Field-specific reporting

Please select the one below that is the best fit for your research. If you are not sure, read the appropriate sections before making your selection.

[x] Life sciences   [ ] Behavioural & social sciences   [ ] Ecological, evolutionary & environmental sciences

For a reference copy of the document with all sections, see nature.com/documents/nr-reporting-summary-flat.pdf

Life sciences study design

All studies must disclose on these points even when the disclosure is negative.

Sample size	1, The fresh leaves of a C. chachiensis tree was used for Nanopore sequencing, whole-genome shotgun sequencing, and Hi-C sequencing, respectively.<br>2, For the transcriptome sequencing, the organs/tissue samples (leaves, flowers, young fruit, peel, pulp, seeds) from different developmental stages of C. chachiensis were collected from the same fruit tree monthly in Xinhui. Three biological replicates for each sample were collected at least.<br>3, For metabolite detection, fruits and peels were collected which both had 3 replicates.<br>4, For TRV-mediated virus-induced gene silencing in citrus fruit, each treatment had three biological replicates with a minimum of 20 citrus fruits.
Data exclusions	For RNA-seq data, the cleaned reads were obtained after removing adapters and filtering low-quality sequences by Trimmomatic (v0.38).
Replication	One plant was used for genome sequencing. The organs and tissue samples (leaves, flowers, young fruits, peels, pulp, seeds) from fruit different developmetal stages for RNA-seq had at least 3 biological replicates. For TRV-mediated virus-induced gene silencing in citrus fruits, each treatment had 3 biological replicates with a minimum of 20 citrus fruits. All attempts were successful.
Randomization	Samples were allocated based on citrus fruits development stages (collected time).
Blinding	Blinding was not applicable.

Reporting for specific materials, systems and methods

We require information from authors about some types of materials, experimental systems and methods used in many studies. Here, indicate whether each material, system or method listed is relevant to your study. If you are not sure if a list item applies to your research, read the appropriate section before selecting a response.

Materials & experimental systems

n/a	Involved in the study	
☒	☐	Antibodies
☒	☐	Eukaryotic cell lines
☒	☐	Palaeontology and archaeology
☒	☐	Animals and other organisms
☒	☐	Clinical data
☒	☐	Dual use research of concern
☐	☒	Plants

Methods

n/a	Involved in the study	
☒	☐	ChIP-seq
☒	☐	Flow cytometry
☒	☐	MRI-based neuroimaging
